# Supplementary material for: Using patient-reported outcome measures during the management of patients with end-stage kidney disease requiring treatment with haemodialysis (PROM-HD): a qualitative study
Source: BMJ Open. 2021 Aug 26;11(8):e052629. doi: 10.1136/bmjopen-2021-052629 (PMC8395280; doi:10.1136/bmjopen-2021-052629)
Supplement: Supplementary data [file bmjopen-2021-052629supp001.pdf]

RRK6261 IRAS ID: 224576

PROM-HD Study

Using patient reported outcome measures (PROMs) to promote quality of care and safety in the management of patients with established kidney disease requiring treatment with haemodialysis

Topic Guide – Patient Participant Interviews

Research Objectives

To explore the feasibility of using routine PROM assessment to aid the management of patients with established renal failure undergoing HD

Respondent Profile

18 years or over, patients who are receiving renal replacement therapy in the form of HD, who meet the study inclusion/exclusion criteria

Logistics

Face-to face interviews: place, date, and time (start and end) of the interviews to be recorded.

Telephone interviews: write the name, title, telephone number of the respondent and record data and time (start and end time) of interview.

Introduction

Introduce self as a Nurse Researcher but explain that whilst I have a nursing background I am not part of their direct healthcare team, so please feel to talk openly and honestly. Participants should be reassured about the confidential nature of the interview and that all comments will be anonymised before use.

Topics and areas of questioning

A: Awareness of PROMs

1. Have you ever completed questionnaires about your QoL or experiences of receiving dialysis before?
2. Having looked at the sample questionnaires – do they capture the things you think are most important about living with kidney disease and being on dialysis
3. Do you think the collection of PROM questionnaires would be beneficial? Do you think the capture of these outcomes is important?

B. Practical Aspects around PROM collection

1. Would you be able to complete a PROM questionnaire successfully on your own, and if not, how much assistance do you think you would require?
2. How often should they be completed and how long should completion take?

Version 2.0 31.10.17

3. Where and when would you want to complete the PROMs i.e. at home? At the Unit? Before or during dialysis?

4. How should PROM data be collected i.e. in paper or electronic formats such as mobile app or telephone recognition systems?

C. Feedback of PROM data

1. How would you like to receive feedback from the clinical team on your PROM data? i.e. email, letter, graphs in person etc.

D. Barriers or facilitators

1. What do you think are the likely factors (barriers and facilitators) that may improve or discourage the completion of PROMs?

2. What do you think are the issues about PROM collection in different patient groups (e.g. older patients, patients of non-white ethnicity)?

E. Use of PROMs in Research

1. Have you have been involved in PROM collection for research purposes?

2. Should PROMs that collect data for routine clinical and research purposes be different?

3. Do you think any of the practical considerations change when you are collecting PROMs for research rather than as part of routine practice? i.e. would you be prepared to complete them more often for research

4. Are there any special considerations to be given to feedback when using PROMs in a research setting?
