# Supplementary material for: Using patient-reported outcome measures during the management of patients with end-stage kidney disease requiring treatment with haemodialysis (PROM-HD): a qualitative study
Source: BMJ Open. 2021 Aug 26;11(8):e052629. doi: 10.1136/bmjopen-2021-052629 (PMC8395280; doi:10.1136/bmjopen-2021-052629)
Supplement: Supplementary data [file bmjopen-2021-052629supp002.pdf]

RRK6261 IRAS ID: 224576

PROM-HD Study

Using patient reported outcome measures (PROMs) to promote quality of care and safety in the management of patients with established kidney disease requiring treatment with haemodialysis

Topic Guide – HCP Interviews

Research Objectives

To explore the feasibility of using routine PROM assessment to aid the management of patients with established renal failure undergoing HD

Respondent Profile

Healthcare Professionals (HCPs) who meet the study inclusion/exclusion criteria.

Logistics

Interview: place, date, and time (start and end) of the interviews to be recorded.

Introduction

Introduce self as a Nurse Researcher but explain that whilst I have a nursing background I am not part of the direct healthcare team, so please feel to talk openly and honestly. Participants should be reassured about the confidential nature of the interview and that all comments will be anonymised before use.

Topics and areas of questioning

A: Awareness of PROMs

1. Have you ever been involved in the collection of PROMs or data regarding QoL and patient experience before?

   Do you think the collection of PROM questionnaires would be beneficial?

   Do you think the capture of these outcomes is important? What do you think about the current measures available and the use of core outcome sets.

   Do you think they could help focus a consultation? Should a 'good' clinician already be able to elicit those outcomes that are important to a patient? Would PROMs alter the way a clinician talks or engages with a patient?

   If yes, how, do you think they could help improve care for a group of patients?

   Do you think they could assist in the multidisciplinary team (MDT) care/follow up of the patient?

   How much time do you spend talking to patients in a typical consultation? Would you like to spend more/less?

2. Having looked at the sample questionnaires – do they capture the things you think are most important about living with kidney disease and being on dialysis from your

perspective as a MDT member?

a. Are there specific aspects of dialysis care that you think would be effected by the use of PROMs

b. How do you think PROMs differ from PREMs? Do you think collection of one is more important than the other?

B. Practical Aspects around PROM collection

1. Do you think most patients would be able to complete a PROM questionnaire successfully on their own, and if not, how much assistance do you think they would require and from whom? Could assistance affect the responses?
2. How often should they be completed and how long should review of the results take?
3. Where and when would you want the PROMs completed i.e. at home? Before or during dialysis?
4. How should PROM data be collected i.e. in paper or electronic formats such as mobile app or telephone recognition systems?

C. Feedback of PROM data

1. Who would be the best member of the MDT to review the data initially? What about alerts? How should they be managed? Some staff said they don’t have time, is that true?
2. What might be the best way the feedback the PROM data to the (a) clinical team, (b) patient? i.e. email, letter, graphs in person etc.
3. How to do you deliver your QA results?
4. How do you feel dealing with problems that you can’t solve – do you feel there are areas outside your area of expertise you would not want to discuss? Is listening enough?
5. Specifically, what PROM data do you think would be important within (a) a clinic environment, (b) an MDT meeting, and how might it be presented? E.g. line graphs, bar charts, raw tabulated data etc.
6. Could they be used to show longitudinal trends? Do you think they would be able to pick up ceiling effects or sensitive changes?

D. Barriers or facilitators

1. What do you think are the likely factors (barriers and facilitators) that may improve or discourage the completion of PROMs? IT issues/time? Importance of leadership during any implementation phase
2. Is there a risk we are going to overburden patients with multiple questionnaires? Have you heard of CAT/item banking?

3. What is the best way to implement into your workflow – could they affect your work flow patterns?

4. Would you want educating about PROMs – what form would that learning take? What specifically would you want more information on? Theory behind collection of PROS, Core outcome sets? Measures available, how they may be used in routine care?
   Are there opportunities to undertake advanced communications training?

5. What do you think about are the key issues about PROM collection in different patient groups (e.g. older patients, patients of non-white ethnicity)?

E. Use of PROMs with patients undergoing Home HD

1. In your opinion, how do patients dialysing at home differ from those dialysing in satellite units? Are they better at 'coping', dealing with change or is it their support structures? Do you think there is utility measuring PROs with this group of patients in particular? Role of carers in this group, how much should they be involved in the process?

2. Would any of your answers differ for patients undergoing Home HD in terms of when, where, how often, who should review and provide feedback?

3. Do you think Home HD patients are more likely to engage with digital technology?

F. Use of PROMs in Research

1. Have you have been involved in PROM use for research purposes? do you know how the measurement tool was chosen? Do you think it captured outcomes that were relevant for the research project?

2. Do you think it would be a good idea to use core outcome sets for renal trials? Should outcomes and the associated PROM that collects them differ between use for routine clinical and research purposes?

3. Do you think any of the practical considerations change when you are collecting PROMs for research rather than as part of routine practice? i.e. how often and where they are completed

4. Are there any special considerations to be given to feedback when using PROMs in a research setting?
