# Supplementary material for: Using patient-reported outcome measures during the management of patients with end-stage kidney disease requiring treatment with haemodialysis (PROM-HD): a qualitative study
Source: BMJ Open. 2021 Aug 26;11(8):e052629. doi: 10.1136/bmjopen-2021-052629 (PMC8395280; doi:10.1136/bmjopen-2021-052629)
Supplement: Supplementary data [file bmjopen-2021-052629supp003.pdf]

Supp Table 1 Patient saturation table and interview characteristics

	Value
**Patient Interviews**	
Number invited/Number participating	41/22
Reasons for non-participation	Did not meet eligibility criteria, did not want to participate (transport issues, did not want to do research)
Duration of patient interviews (min)	Range 24-84<br>Mean 50 mins
**Patient Saturation Data**	
No of codes generated	110
Point of data saturation	15th interview
Note n=17	

		1	2	3	4	5	6	7	8	9	10	11	12	13	14	15	16	17	18	19	20	21	22
**1 Innovation Characteristics**																							
1A Innovation Source													x						x				
1B Evidence strength and quality		/	/	/	/	/	/	/	/	/	/	/	/	/	/	/	/	/	/	/	/	/	/
1C Relative Advantage	Perception of advantage	x	x		x	x	x	x			x	x	x		x	x	x		x		x	x	
	Intractable problems								x			x		x	x	x					x		
	Potential to cause anxiety										x			x	x		x			x	x	x	
1D Adaptability	Adaptability of intervention												x						x				
	Use within research	x	x	x	x			x	x			x	x	x		x	x		x				
	Importance of choices in how and what is answered	x	x	x				x					x				x	x				x	
1E Trialability		/	/	/	/	/	/	/	/	/	/	/	/	/	/	/	/	/	/	/	/	/	/
IF Complexity								x											x				
IG Design and Packaging	Frequency	x	x		x		x	x		x	x	x		x	x	x	x			x		x	x
	Length	x	x	x	x		x	x		x	x	x	x	x	x	x	x	x	x	x	x		x
	Language/Readability					x		x	x	x	x	x	x		x		x	x			x		x
	Mode	x	x	x	x	x	x	x	x	x	x	x		x	x	x	x	x	x	x	x	x	x

Supp Table 1 Patient saturation table and interview characteristics

		1	2	3	4	5	6	7	8	9	10	11	12	13	14	15	16	17	18	19	20	21	22
	Setting	x		x	x	x		x	x		x	x	x	x		x	x	x		x	x	x	x
	Timing	x	x		x	x	x		x	x	x			x	x	x		x	x	x		x	x
	Outcomes that matter	x	x		x	x	x			x			x	x	x	x			x	x		x	
	Current Measures				x	x	x	x	x		x	x	x	x	x		x	x	x		x	x	x
	Safety Alerts	x																	x			x	
	Self-help information		x						x				x	x			x					x	
	ComputerAdaptiveTechnology										x	x		x		x	x		x	x			
	Review and Feedback	x	x		x		x	x	x	x	/	x	x	/	x	x	/	x	x	x		x	x
	Sensitivity to change	/	/	/	/	/	/	/	/	/		/	/		/	/		/	/	/	/	/	/
IH Cost																			x				
**2 Outer Setting**																							
2A Needs and resources	Identification		x	x	x		x	x	x			x	x	x	x			x	x	x	x	x	x
	Barriers	x	x	x	x	x	x	x	x	x	x	x	x	x	x	x	x	x	x	x	x	x	x
	Facilitators	x	x	x	x		x			x	x	x	x	x				x	x	x		x	
2B Cosmopolitanism				x				x			x				x					x			
2C Peer Pressure		/	/	/	/	/	/	/	/	/	/	/	/	/	/	/	/	/	/	/	/	/	/
2D External policies and incentives		/	/	/	/	/	/	/	/	/	/	/	/	/	/	/	/	/	/	/	/	/	/
**3 Inner Setting**																							
3A Structural Characteristics																x							
3B Networks and Communications	Nature and Quality		x	x	x	x	x	x	x	x	x	x	x		x	x		x	x	x	x	x	x
	Shared Decision Making	x	x	x							x	x	x	x	x	x		x		x	x		x
3C Culture											x			x	x	x					x		
3D Implementation Climate	Tension for change		x	x					x			x	x	x	x			x	x	x	x		x
	Compatibility							x				x	x									x	x
	Relative Priority	x			x						x										x	x	
	Organisational Incentives	/	/	/	/	/	/	/	/	/	/	/	/	/	/	/	/	/	/	/	/	/	/

Supp Table 1 Patient saturation table and interview characteristics

		1	2	3	4	5	6	7	8	9	10	11	12	13	14	15	16	17	18	19	20	21	22
	Goals and Feedback	/	/	/	/	/	/	/	/	/	/	/	/	/	/	/	/	/	/	/	/	/	/
	Learning Climate												x										
3E Readiness for Implementation	Leadership Engagement	/	/	/	/	/	/	/	/	/	/	/	/	/	/	/	/	/	/	/	/	/	/
	Available Resources		x		x											x		x				x	
	Access to knowledge and info		x			x	x						x		x	x	x				x	x	x
**4 Characteristics of Individuals**																							
Knowledge and beliefs		x	x	x	x			x	x	x		x		x	x	x	x	x	x	x	x	x	
Self-efficacy	Ability to self-complete	x	x	x		x	x	x	x	x	x	x	x	x	x	x	x	x		x		x	
	Patient as an Expert			x				x				x	x	x	x					x			
	PROMs v PREMs		x		x	x		x											x		x		
Individual stage of change	Previous experience of PROMs usage	x			x	x	x		x	x	x	x	x	x	x	x	x	x	x	x	x	x	x
Individual identification with organisation		x	x		x	x	x	x	x	x	x	x	x	x	x		x			x	x	x	x
Other personal attributes	Multidimensionality of ESKD:																						
	Symptomology	x	x	x	x	x	x	x	x	x	x	x	x	x	x	x	x	x	x	x	x	x	x
	Life on Dialysis	x	x	x	x	x	x	x	x	x	x	x	x	x	x	x	x	x	x	x	x	x	x
**5 Process**																							
5A Planning		/	/	/	/	/	/	/	/	/	/	/	/	/	/	/	/	/	/	/	/	/	/
5B Engaging																							
	Opinion Leaders	/	/	/	/	/	/	/	/	/	/	/	/	/	/	/	/	/	/	/	/	/	/
	Formally appointed leaders					x		x															
	Champions					x							x	x									
	External Change Agents	/	/	/	/	/		/	/	/	/	/	/	/	/	/	/	/	/	/	/	/	/
	Key Stakeholders	/	/	/	/	/		/	/	/	/	/	/	/	/	/	/	/	/	/	/	/	/
	Innovation Participants				x								x										
	Role of Carers	x		x	x		x	x		x			x				x						
5C Executing		/	/	/	/	/	/	/	/	/	/	/	/	/	/	/	/	/	/	/	/	/	/
5D Reflecting and Evaluating		/	/	/	/	/	/	/	/	/	/	/	/	/	/	/	/	/	/	/	/	/	/

BMJ Publishing Group Limited (BMJ) disclaims all liability and responsibility arising from any reliance placed on this supplemental material which has been supplied by the author(s)

Supp Table 1 Patient saturation table and interview characteristics

First time data coded to node in NVIVO	x
Nothing coded to node in NVIVO	/
Nothing coded to node in NVIVO	/
