# Supplementary material for: Using patient-reported outcome measures during the management of patients with end-stage kidney disease requiring treatment with haemodialysis (PROM-HD): a qualitative study
Source: BMJ Open. 2021 Aug 26;11(8):e052629. doi: 10.1136/bmjopen-2021-052629 (PMC8395280; doi:10.1136/bmjopen-2021-052629)
Supplement: Supplementary data [file bmjopen-2021-052629supp004.pdf]

Suppl Table 2 Healthcare Professional Participants: Saturation table and interview characteristics

	Value
**HCP Interviews**	
Number invited/Number participating	19/17
Reasons for non-participation	Competing clinical commitments
Duration of patient interviews (min)	Range 29-63<br>Mean 48 mins
**Patient Saturation Data**	
No of codes generated	134
Point of data saturation	16th Interview
Note n=17	

**Participants n=17**		**1**	**2**	**3**	**4**	**5**	**6**	**7**	**8**	**9**	**10**	**11**	**12**	**13**	**14**	**15**	**16**	**17**
**1 Innovation Characteristics**																		
1A Innovation Source						x										x		
1B Evidence strength and quality		x				x				x		x		x	x	x	x	x
1C Relative Advantage		x	x	x	x	x	x		x	x	x	x	x	x	x	x	x	x
												x	x		x		x	x
	How PROMs might work to deliver advantage		x		x	x	x	x	x	x								
1D Adaptability		x				x												x
	Use within research				x	x							x		x	x	x	
1E Trialability																	x	
IF Complexity		x				x	x		x		x						x	
IG Design and Packaging		x	x	x	x	x	x	x	x	x	x	x	x	x	x	x	x	x
	Frequency	x	x	x	x	x	x	x	x	x	x	x	x	x	x	x		x
	Length			x	x	x	x		x		x	x	x	x	x		x	
	Mode	x	x		x	x			x	x	x	x	x	x		x	x	

Suppl Table 2 Healthcare Professional Participants: Saturation table and interview characteristics

**Participants n=17**		**1**	**2**	**3**	**4**	**5**	**6**	**7**	**8**	**9**	**10**	**11**	**12**	**13**	**14**	**15**	**16**	**17**
	Setting		x	x	x	x		x	x	x	x			x			x	x
	Timing		x	x	x		x		x	x	x	x	x	x	x		x	
	Self-completion		x	x	x	x	x	x				x	x	x	x	x		x
	Outcomes that matter	x	x	x	x		x		x	x	x		x			x		x
	Safety Alerts			x			x				x			x	x	x	x	
	Sensitivity						x		x		x	x						
	Computer Adaptive Technology					x		x	x	x	x	x	x	x		x	x	
	Review and Feedback	x	x	x	x	x	x	x				x	x	x	x	x	x	x
IH Cost														x		x		
**2 Outer Setting**																		
2A Needs and resources						x	x	x		x	x	x	x	x		x		
	Barriers		x		x	x	x	x	x	x	x	x	x	x	x	x	x	x
	Facilitators			x	x	x			x		x	x	x				x	
2B Cosmopolitanism				x	x		x	x					x					
2C Peer Pressure																		
2D External policies and incentives				x		x	x	x	x					x	x		x	x
**3 Inner Setting**																		
3A Structural Characteristics		x	x	x	x		x	x				x	x	x	x			
3B Networks and Communications		x		x	x	x		x	x	x	x	x	x		x	x		x
3C Culture		x	x	x		x	x	x			x	x	x			x	x	x
3D Implementation Climate																		
	Tension for change	x	x	x		x			x	x	x			x				
	Task focused approach to care							x	x			x	x		x	x	x	x
	Compatibility	x				x	x		x		x	x					x	
	Relative Priority	x					x								x			
	Organisational Incentives																	
	Goals and Feedback		x			x				x								
	Learning Climate		x	x								x						x
3E Readiness for Implementation	Leadership Engagement		x									x						

Suppl Table 2 Healthcare Professional Participants: Saturation table and interview characteristics

**Participants n=17**		**1**	**2**	**3**	**4**	**5**	**6**	**7**	**8**	**9**	**10**	**11**	**12**	**13**	**14**	**15**	**16**	**17**
	Available Resources	x	x	x	x	x	x	x	x	x	x	x	x	x	x	x	x	x
	Access to knowledge and info		x	x	x	x		x	x	x	x		x				x	x
**4 Characteristics of Individuals**																		
Knowledge and beliefs		x	x	x		x		x	x	x			x		x		x	x
Self-efficacy		x	x	x		x	x		x	x	x		x		x	x		
	Ability of patients to self-complete	x	x	x	x	x	x	x	x	x	x	x	x	x	x	x	x	x
Individual stage of change	Previous experience of PROMs usage	x	x	x	x	x	x	x	x	x	x		x	x	x	x	x	
Individual identification with organisation			x	x						x	x		x					
Other personal attributes		x	x	x	x		x	x				x	x	x	x	x	x	
**5 Process**																		
5A Planning						x		x										
5B Engaging						x	x		x									x
	Opinion Leaders	x				x	x				x							x
	Formally appointed leaders	x		x	x	x		x	x	x	x		x		x			x
	Champions		x	x				x			x		x					x
	External Change Agents												x			x		x
	Key Stakeholders					x												
	Innovation Participants					x												
5C Executing																		
5D Reflecting and Evaluating																x		

First time data coded to this node in NVIVO	x
Nothing coded to this node in NVIVO	
