# Supplementary material for: Using patient-reported outcome measures during the management of patients with end-stage kidney disease requiring treatment with haemodialysis (PROM-HD): a qualitative study
Source: BMJ Open. 2021 Aug 26;11(8):e052629. doi: 10.1136/bmjopen-2021-052629 (PMC8395280; doi:10.1136/bmjopen-2021-052629)
Supplement: Author's manuscript [file bmjopen-2021-052629.draft_revisions.pdf]

BMJ Open is committed to open peer review. As part of this commitment we make the peer review history of every article we publish publicly available.

When an article is published we post the peer reviewers' comments and the authors' responses online. We also post the versions of the paper that were used during peer review. These are the versions that the peer review comments apply to.

The versions of the paper that follow are the versions that were submitted during the peer review process. They are not the versions of record or the final published versions. They should not be cited or distributed as the published version of this manuscript.

BMJ Open is an open access journal and the full, final, typeset and author-corrected version of record of the manuscript is available on our site with no access controls, subscription charges or pay-per-view fees (http://bmjopen.bmj.com).

If you have any questions on BMJ Open's open peer review process please email
info.bmjopen@bmj.com

BMJ Open

Using Patient Reported Outcome Measures during the management of patients with end stage kidney disease requiring treatment with haemodialysis (PROM-HD): a qualitative study.

Journal:	*BMJ Open*
Manuscript ID	bmjopen-2021-052629
Article Type:	Original research
Date Submitted by the Author:	27-Apr-2021
Complete List of Authors:	Anderson, Nicola; University of Birmingham; University Hospitals Birmingham NHS Foundation Trust<br>McMullan, Christel; University of Birmingham, Institute of Applied Health Research<br>Calvert , Melanie ; University of Birmingham, Centre for Patient Reported Outcomes Research and Institute of Applied Health Research<br>Dutton, Mary; University Hospitals Birmingham NHS Foundation Trust, Renal Medicine<br>Cockwell, Paul; University Hospitals Birmingham NHS Foundation Trust, Department of Renal Medicine<br>Aiyegbusi, Olalekan ; University of Birmingham, Centre for Patient-Reported Outcomes Research; University of Birmingham, NIHR Birmingham Biomedical Research Centre, University of Birmingham and University Hospitals Birmingham NHS Foundation Trust<br>Kyte, Derek; University of Worcester
Keywords:	NEPHROLOGY, End stage renal failure < NEPHROLOGY, QUALITATIVE RESEARCH

SCHOLARONE™
Manuscripts

I, the Submitting Author has the right to grant and does grant on behalf of all authors of the Work (as defined in the below author licence), an exclusive licence and/or a non-exclusive licence for contributions from authors who are: i) UK Crown employees; ii) where BMJ has agreed a CC-BY licence shall apply, and/or iii) in accordance with the terms applicable for US Federal Government officers or employees acting as part of their official duties; on a worldwide, perpetual, irrevocable, royalty-free basis to BMJ Publishing Group Ltd ("BMJ") its licensees and where the relevant Journal is co-owned by BMJ to the co-owners of the Journal, to publish the Work in this journal and any other BMJ products and to exploit all rights, as set out in our licence.

The Submitting Author accepts and understands that any supply made under these terms is made by BMJ to the Submitting Author unless you are acting as an employee on behalf of your employer or a postgraduate student of an affiliated institution which is paying any applicable article publishing charge ("APC") for Open Access articles. Where the Submitting Author wishes to make the Work available on an Open Access basis (and intends to pay the relevant APC), the terms of reuse of such Open Access shall be governed by a Creative Commons licence – details of these licences and which Creative Commons licence will apply to this Work are set out in our licence referred to above.

Other than as permitted in any relevant BMJ Author's Self Archiving Policies, I confirm this Work has not been accepted for publication elsewhere, is not being considered for publication elsewhere and does not duplicate material already published. I confirm all authors consent to publication of this Work and authorise the granting of this licence.

For peer review only

Qualitative Study – Manuscript

Using Patient Reported Outcome Measures during the management of patients with end stage kidney disease requiring treatment with haemodialysis (PROM-HD): a qualitative study.

Nicola Anderson MsC[1,2], Christel McMullan Phd[1,3], Melanie Calvert PhD[1,3,4,6,7], Mary Dutton MSc[2], Paul Cockwell PhD[5], Olalekan L Aiyegbusi PhD [1,4,6,7] Derek Kyte PhD[1,4]

[1] Institute of Applied Health Research, Centre for Patient Reported Outcomes Research, University of Birmingham, Birmingham, United Kingdom

[2]Research and Development, University Hospitals Birmingham NHS Foundation Trust

[3] NIHR SRMRC, University Hospitals Birmingham NHS Foundation Trust and University of Birmingham, UK

[4] NIHR Birmingham Biomedical Research Centre, University Hospitals Birmingham NHS Foundation Trust and University of Birmingham, UK

[5] Department of Nephrology, University Hospitals Birmingham NHS Foundation Trust

[6] Birmingham Health Partners Centre for Regulatory Science and Innovation

[7] NIHR Applied Research Collaboration West Midlands, University of Birmingham

Corresponding author: Nicola Anderson NEA451@bham.ac.uk

For peer review only - http://bmjopen.bmj.com/site/about/guidelines.xhtml

ABSTRACT

Rationale

Patients undergoing haemodialysis report elevated symptom burden and reduced health-related quality of life and often prioritise improvements in psychosocial wellbeing over long-term survival. Systematic collection and use of patient reported outcomes (PROs), particularly via electronic means, may help support tailored healthcare and improve outcomes. This study explored perceptions and experiences of patients receiving haemodialysis and Healthcare Professionals (HCPs) on implementation and use of PROs in routine clinical practice.

Study design

Qualitative study

Setting and participants

Semi structured interviews with 22 patients undergoing haemodialysis, and 17 Healthcare Professionals in the United Kingdom.

Analytical Approach

Transcripts were analysed deductively using the Consolidated Framework for Implementation Research (CFIR) and inductively using Thematic Analysis.

Results

For effective implementation, the potential benefits of PROs need to be demonstrated empirically to both patients and Healthcare Professionals. Any intervention must remain flexible enough for both individual and aggregate use, whilst maintaining operational simplicity.

For peer review only

For peer review only - http://bmjopen.bmj.com/site/about/guidelines.xhtml

It is important that outcomes that matter to patients are measured, whilst recognising the potential of PROs to create efficiencies and improvements in healthcare delivery.

A possible incompatibility of PROs and approaches to delivery of care in HD settings was identified, along with recognition that any implementation must sit within a wider framework of education and support for both patients and clinicians who demonstrate varying previous experience of using PROs and often confuse related concepts. Implementation plans must recognise the multidimensionality of end stage kidney disease and the associated challenges of complex heath needs in a highly specialised environment.

To support implementation, careful consideration needs to be given to barriers and facilitators including effective leadership, the role of champions, effective launch, and ongoing evaluation.

Conclusions

The findings of this study can assist PRO implementation plans, by identifying the priorities and concerns of both patients and clinicians, including timely understanding of facilitators and barriers.

Key Words:

Haemodialysis, End Stage Kidney Disease, Patients Reported Outcomes, Electronic Patient Reported Outcomes, Health Related Quality of Life

For peer review only

For peer review only - http://bmjopen.bmj.com/site/about/guidelines.xhtml

Article Summary

Strengths and limitations of this study

- Qualitative methods yield large amount of rich data with face to face semi-structured interviews allowing the interviewer to monitor non-verbal communications and clarify ambiguous responses.

- Large amounts of qualitative data mean the analysis phase is time consuming. A framework approach and qualitative coding software was used to assist this process.

- The role of the researchers was carefully considered, to acknowledge and minimise bias associated with beliefs and values. Steps were taken to mitigate risks, including use of reflective diaries, participant checking and multiple researchers involved in the coding process.

- While purposive sampling methods led to a diverse sample of participants, it is acknowledged that the sample did not include non-English speakers or carers. Further research is required.

- Data was collected before the coronavirus pandemic. The healthcare delivery landscape in the UK has changed, and it is possible that some attitudes and beliefs particularly around digital data capture may have evolved.

For peer review only - http://bmjopen.bmj.com/site/about/guidelines.xhtml

Funding Statement

This presentation refers to independent research funded by the National Institute of Health Research (NIHR) under its Clinical Doctoral Research Fellowship Programme (Grant Reference ICA-CDRF-2018-04-ST2-027)

Competing interests

MC is a NIHR Senior Investigator and receives funding from the NIHR Birmingham Biomedical Research Centre, the NIHR Surgical Reconstruction and Microbiology Research Centre and NIHR Applied Research Collaboration West Midlands at the University of Birmingham and University Hospitals Birmingham NHS Foundation Trust, Health Data Research UK, Innovate UK (part of UK Research and Innovation), Macmillan Cancer Support, UCB Pharma and GSK. MC has received personal fees from Astellas, Takeda, Merck, Daiichi Sankyo, Glaukos, GSK and the Patient-Centered Outcomes Research Institute (PCORI) outside the submitted work. DK reports grants from Macmillan Cancer Support, Innovate UK, the NIHR, NIHR Birmingham Biomedical Research Centre, and NIHR SRMRC at the University of Birmingham and University Hospitals Birmingham NHS Foundation Trust, and personal fees from Merck and GSK outside the submitted work. O.L.A. is funded by the NIHR BRC, Birmingham and declares personal fees from Gilead Sciences Ltd. and GSK. The remaining authors declare no competing interests.

The views expressed are those of the author(s) and not necessarily those of the NHS, the NIHR or the Department of Health. The study sponsor and funders have no role in study design, including collection, management, analysis and interpretation of data; writing of the report and the decision to submit the report for publication.

For peer review only

INTRODUCTION

The prevalence of end stage kidney disease (ESKD) requiring treatment with renal replacement therapies such as haemodialysis (HD) continues to rise worldwide (1). Both underlying disease and treatment are associated with a high symptom burden and reduced health-related quality of life (HRQoL) (2, 3). Historically, outcomes such as mortality and dialysis adequacy based on biomedical parameters have been used to inform the management of dialysis services and individual care (4). However, there is now a body of established research (5-8) (9, 10) demonstrating the importance of also capturing patient-reported outcomes (PROs). PROs capture data directly from patients on how they feel or function, without requiring interpretation from others (11). There is also much interest in the role of electronic PRO collection (ePROs) to support remote symptom monitoring in HD (12). Findings from studies in oncology have demonstrated the potential benefits of routine ePRO capture, including improvements in HRQoL, symptom control and overall survival (13).

Whilst patient-reported experience measures (PREMs) have been collected in the UK since 2017 (14), the use of electronic capture is sparse and PROs are still not routinely and systematically collected to manage individual patient care (15).

Before routine PROs collection is adopted in the UK, it is important to explore the views and experiences of patients receiving HD and Healthcare Professionals (HCPs) on their overall purpose and implementation. Therefore, the aim of this project was to conduct a qualitative study to examine the acceptability and feasibility of routine PRO assessment in HD settings.

METHODS

Design: The methodological orientation underpinning this study is pragmatism and accordingly, the research question is the major determinant of the method. The study explores

For peer review only - http://bmjopen.bmj.com/site/about/guidelines.xhtml

personal experiences, values and beliefs, phenomena that are difficult to quantify, therefore a study employing qualitative methodology was designed.

Participant Selection:

Ethics approval was issued on 13 September 2017 (Ref No: 17/WA/0281).

Participants were identified and recruited between July 2018 and November 2019. Eligible patients were primarily approached face to face in the dialysis unit. They included adults (≥18years), receiving HD (in-centre or at home) able to provide valid informed consent and converse in everyday English. Patients were excluded if they were not deemed established on HD or had been undergoing HD < 3 months, or if they had an active intercurrent medical problem requiring enhanced routine clinical care (16). Purposive sampling was undertaken to achieve maximum variation across age, gender, ethnicity, time on dialysis and co-morbidities (17). HCPs were recruited from the broader renal team, and initially contacted by email, they included Health Care and Administrative Assistants (16).

Data Collection:

Data was collected from 22 patients undergoing haemodialysis, and 17 Healthcare Professionals in the United Kingdom: All consenting participants took part in an audio-recorded semi-structured interview with the lead author, either face-to-face or by telephone. Ethical approval was gained for follow up interviews, but none were required. Patient interviews were conducted in the dialysis unit for those dialysing in-centre and at home for the Home HD group. Since interviews were conducted in patient homes, family members were sometimes present at the request of the patient, but did not take an active role in the interview. HCP's were interviewed in a private room at their workplace. Topic guides were used to steer and support the interview process (see Appendices). These guides were piloted with one participant from each group and refined iteratively during the collection phase in

For peer review only - http://bmjopen.bmj.com/site/about/guidelines.xhtml

response to initial findings. Field notes and in-depth memos were created after each interaction. Since PROs are not routinely collected in the UK, it was recognised pre-interview information was required to aid the quality of discussion. Therefore participants were provided with a diagram of a core outcome set selected specifically for use in HD trials, to illustrate the position of PROs in outcome measurement (18) and example PROs from a recent systematic review and supported by our local Renal Public and Patient Involvement group: (Kidney Disease Quality of Life SF (KDQOL-SF) (19), Kidney Disease Quality of Life 36 (KDQOL-36) (20) and Integrated Patient Outcome Scale – Renal (IPOS-Renal)) (21).

Analysis

Using Codebook Thematic Analysis (22), a coding framework, drawing upon the Consolidated Framework for Implementation Research (CFIR) (23), was used to deductively analyse transcripts, with further new subthemes developed inductively through data engagement and the analytic process.

The CFIR is a widely utilised conceptual framework developed to guide systematic assessment of factors that might influence implementation and effectiveness (See Figure 1) (23, 24). Primary data analysis was conducted by the lead author, with two investigators (CMcM, DK) reviewing coding for consistency and appropriateness. As the lead author was a renal research nurse conducting this research as part of a Clinical Doctoral Research Fellowship, she was known to some HCP participants. This information was declared and discussed during the valid informed consent process. Additionally a reflective research diary, memo-writing and discussion with the study management team were used to try to minimise the influence of prior relationships upon analysis. Data analysis was supported by Qualitative Data Analysis Software – NVIVO 12 QSR International. Participant verification was undertaken on one transcript from each group – no discrepant comments were reported. Data

For peer review only - http://bmjopen.bmj.com/site/about/guidelines.xhtml

collection and analysis continued until saturation was achieved, i.e. no new information pertinent to the research question was being generated by further interviews (see Suppl Saturation Tables 1 and 2) (25).

Patient and Public Involvement

Research on the use and implementation of PROs in nephrology settings was prioritised by the local Kidney Patients Charity. An existing PPI group was utilised and a study specific PPI group was convened to help develop the research question, design this study and the associated Fellowship application. Patients were consulted on the study documentation, including topic guides and supplementary materials. A summary of study findings will be made available for study participants.

RESULTS

Participant characteristics

Participant characteristics are summarised in Tables 1 and 2.

The patient sample was broadly representative of prevalent HD population in the UK (27) . All participants were being treated via, or working at, a large regional hospital. To accurately reflect the diversity within the dialysis population, patients were recruited from two satellite units and those patients choosing to dialyse at home. Non-medical members of the multidisciplinary team were targeted from these two units, one in a city and one serving mainly rural communities. All HCPs working in the home setting had extensive previous experience of in-centre dialysis delivery.

Data Saturation was deemed achieved after interviewing 16 HCPs and 15 patients (see supplementary tables 1 and 2 for further detail).

For peer review only - http://bmjopen.bmj.com/site/about/guidelines.xhtml

Key Findings

Analysis identified the following themes, which are presented in line with the CFIR key domains. Although presented in a linear fashion, the five domains and their respective constructs cannot be considered in isolation, all interact to effect implementation.

Figure 1 shows the conceptual framework and associated findings. Illustrative quotations are provided in Box 1.

1. Intervention Characteristics: Factors associated with design and quality of the intervention, including how intervention is perceived.

The importance of demonstrating PRO efficacy and impact

Both patients and HCP participants felt that PROs could support the delivery of person-centred care through shared decision making and management in dialysis settings. However, HCP interviewees highlighted the key challenge was not the physical collection of PROs, but how the data was then used to improve outcomes. For patients, it was key that any time spent completing questionnaires should be rewarded with review and appropriate action, i.e., not a ‘tick-box’ exercise.

HCPs, particularly nephrologists, extolled the importance of PROs in research settings.

Intervention flexibility

The topic guide initiated discussions with participants on key practical considerations, including frequency (of PRO completion), optimal timing (around dialysis), preferred setting (home or in-centre), favoured mode of administration (electronic or paper versions) and interpretation and feedback of the responses.

For peer review only - http://bmjopen.bmj.com/site/about/guidelines.xhtml

It was clear from patients that a fixed means of implementing PROs would not meet the requirements of this heterogeneous group. Any system would need to be flexible whilst maintaining maximum simplicity. Most patients felt they could complete electronic measures if required, but some strongly favoured paper options. Most interviewees stated that self-completion was possible but felt some would need physical or emotional support from carers. A few patients indicated a desire for assistance with IT aspects until they became familiar. Several patients alluded to the need to 'compartmentalise' their dialysis, by conducting all dialysis-related activity in-centre or by having discrete facilities and times for dialysis if being treated at home; suggesting that PRO completion would need to be undertaken at defined times/settings within their schedule. Others were less concerned about daily reminders of their disease and management, so were open to completion timings/settings that were more variable.

HCPs discussed the promise of electronic capture to support enhanced management of chronic symptoms, but some expressed concerns about potentially missing acute signs and effectiveness of associated safety reporting and actions. Two Home HD patients foresaw the potential benefits of automated safety alerts associated with electronic capture. However, one patient noted that it was important that any automated information or self-help advice should not conflict with information given face to face by the doctor.

Participants were asked for their perspectives on Computer Adaptive Technology (CAT), a type of assessment in which questions are generated specifically for each individual, using Item Response Theory. This was a new concept to nearly all participants, but most quickly grasped the underlying theory after short explanation and were supportive of the idea, recognising the potential to stop redundant questions and save time. (see Box 2 for overview of practical considerations).

For peer review only - http://bmjopen.bmj.com/site/about/guidelines.xhtml

2. Outer Setting: the wider societal, economic, and organisational context in which the stakeholders and organisation implementing the intervention reside.

Identifying outcomes that matter

There was general agreement amongst all participants that it was important to ensure measurement of ‘what outcomes matter most’ to patients (28). There was broad agreement from patients that the example PROs they had been provided with were comprehensive in covering the key symptoms facing patients undertaking HD. Furthermore, they agreed with findings from the SONG-HD initiative (18) that fluctuations in HRQoL mattered more, to them, than biomedical outcomes. The importance of these outcomes were acknowledged by the HCPs, but it was felt that deep exploration of some PRO data fell outside the remit of the nephrologist or dialysis nurse and there was anxiety about acting outside their competency and the associated risks of litigation.

The ’Bigger Picture’

Several patients highlighted problems with continuity of care and lack of cohesion between primary care and their dialysis provision. One Home HD patient felt the use of PROs could assist uptake of home therapies, by demonstrating better overall outcomes/HRQoL for patients managing their own dialysis. Participants in both groups reflected that PROs could help support shared decision making by targeting and prioritising discussions according to the patient’s agenda.

3. Inner Setting: the structural and cultural contexts around where the implementation will take place

Compatibility of PROs use and approaches to care in HD settings.

For peer review only - http://bmjopen.bmj.com/site/about/guidelines.xhtml

Discussing potential implementation and how participants perceive the current situation as needing change, patients and staff alike discussed the approaches to care within dialysis settings. Patients clearly described a 'task orientated' style of care in-centre, with a focus on practically administering HD, versus a more patient-centred model discussed by those dialysing at home. Patients dialysing themselves felt they had developed a level of expertise and the increased 'control' had improved their HRQoL. They indicated current communication pathways were effective and that the Home HD team was responsive and holistic in their approach to care. Analysis of patient and HCP data suggested that time constraints were the major reason for a task-based approach in-centre with both patients and staff exhibiting a desire to complete dialysis sessions with as little impediment as possible. Correspondingly most in-centre patients did not feel staff had time to have long discussions and some suggested PROs might help rectify this. There was no discernible variation in the views of the Home HD cohort on the practicalities of PRO collection.

Part of a wider framework: The 'information loop', the role of PROs in addressing training needs and health literacy

HCP data analysis highlighted the importance of education, training, and support for successful implementation. It was indicated that while medical staff needed training in PRO interpretation, such as rating scales, nurses might need a broader program on use of PROs as well as associated chronic disease management. There was disparity indicated in the health literacy of the patient group; some Home HD patients appeared expert in their illness and treatment, whilst some in-centre patients appeared less confident in their knowledge.

4. Characteristics of Individuals: Factors associated with individuals involved in implementation

Previous experience of using PROs

For peer review only - http://bmjopen.bmj.com/site/about/guidelines.xhtml

Patients expressed an overall lack of awareness and experience of PROs. Many had completed annual PREMs but highlighted a lack of feedback or action. Some patients expressed general anxiety about questionnaire completion, linked to their experience of externally administered questionnaires for other agencies: for example, Personal Independence Payment (PIP) surveys assessing entitlement to extra costs associated with long term ill health or disability. When reviewing the example PROs, it was noted that some patients, particularly those from minority ethnic backgrounds, flagged the potential difficulty around asking about sensitive issues such as sexual function. Whilst the participants themselves were happy to discuss such issues, they recognised that not all patients might be.

All nephrologists interviewed had experience of using PROs in research settings, but none had regularly and systematically collected PROs in routine care. PROs use in any setting was largely a new concept for nursing staff.

There was confusion around the concepts of PROs versus PREMs, expressed by both patients and HCPs. Participants found it hard to distinguish experience of care from outcomes and used terms interchangeably.

Multidimensional aspects of ESKD and consequent challenges

Many of the patients in the study had multiple co-morbidities and associated symptoms, reflective of the wider HD population. Patients indicated that it was often hard to know which symptoms related to their ESKD and/or treatment, and which were associated with other diseases or advancing age; leading to anxiety about what to raise during a consultation. It was suggested that PROs data review and feedback gave a chance to discuss symptoms and potential causes, thereby potentially providing reassurance. However, some clinicians thought it was outside their role to manage non-nephrology related outcomes presented by PROs and that it would be difficult to disentangle several, possibly unrelated, symptoms. They feared

For peer review only - http://bmjopen.bmj.com/site/about/guidelines.xhtml

focusing on symptoms that were not currently being experienced or intractable could cause frustration or anxiety for themselves and patients. Some patients gave a contrasting opinion, revealing the chance to be heard, even without a solution, was often sufficient to maintain the patient-clinician relationship. There was also concern that an over reliance on PROs could result in a distraction from other important clinical issues.

The experience of life on dialysis was highly varied, with some participants exhibiting signs of depression, anger, or acceptance, as well as reporting multiple symptoms.

5. The Process of Implementation

Supporting the process of implementation

The analysis identified leadership, the identification of champions and a 'bottom up' approach to communication and shared solutions among both patients and clinicians as key factors supporting implementation. All participants emphasised the importance of support during completion, however, patients highlighted this need not always be delivered by health care staff. Peer-to-peer support and non-clinical champions i.e., administrative staff, might assist. HCPs felt a comprehensive launch was important and that individual roles should be clear. They reflected that senior members of the clinical team would be opinion leaders, and any change agents would need to understand the dialysis setting. Evaluation and reflection were important process components, to recognise and deal with any unintended consequences.

Overarching themes: Barriers and Facilitators to effective implementation

Across all CFIR constructs, analysis identified potential barriers and facilitators to the introduction of PROs.

Potential barriers

For peer review only - http://bmjopen.bmj.com/site/about/guidelines.xhtml

Nephrologists cited a lack of evidence base supporting the use of PROs in routine kidney care and were concerned regarding the risk of over-medicalising the patient experience. There were perceived time barriers for staff i.e., workflow interruptions, additional obligations caused by PROs; with nurses perceiving quality time with patients in-centre as limited. HCP participants argued that nursing documentation had lessened available time, as it often required recording on digital devices away from the bedside. They feared patients were being overburdened by questionnaires, particularly the less health literate.

In contrast, patients were often already aware of the complications associated with ESKD and HD. Many had achieved a degree of acceptance and some were reassured that the symptoms were expected and not something new to deal with. Frustration would only arise if, having taken time to complete questionnaires, no action was taken. Both groups agreed that information technology issues i.e., inability to log in, no Wi-Fi could be a barrier.

Potential facilitators

Several HCPs considered how PROs might work to deliver a relative advantage over current systems to support more patient-centred care. Participants felt PROs could be a communication tool – acting like a 'tin opener'; but requiring that responses be carefully probed, highlighting that this required appropriate skills and training. Others saw the measures as an Aide Memoire which could help target the consultation and even save time; as could the possibility of remote management of care using ePROs. Perhaps most importantly, PROs were seen by both patient and HCPs as a way to get to know the patient and hence deliver more meaningful care.

DISCUSSION

For peer review only - http://bmjopen.bmj.com/site/about/guidelines.xhtml

This is the first paper which focuses on PROs and the related experiences and perceptions of patients undergoing HD and the HCPs caring for them. Previous studies have included non-dialysis dependent Chronic Kidney Disease (CKD) populations and nephrologists (29, 30).

The key findings of this study are, that while patients and HCPs support the concept of PROs, further evidence of their potential benefit are required for effective implementation. It emphasises that any system should be flexible and measure what matters most to patients. Most importantly the data collected should be acted upon. There was a general lack of awareness and experience of PROs particularly among patients and nurses, with concern amongst the HCPs that PRO capture may highlight issues they might have neither the experience nor resources to manage. Therefore, a comprehensive implementation strategy is required to support any delivery, which involves strong leadership, patient and clinician involvement and ongoing training.

This study highlighted the importance of getting 'buy in' i.e., gaining acceptability, from these stakeholders. This could be achieved by demonstrating evidence on their potential benefits thereby increasing trust to warrant practice change. Clinicians particularly questioned whether positive effects on survival, reported QoL, patient-clinician communications and cost efficiencies demonstrated in oncology (13, 31-33) could be replicated in HD settings.

A body of evidence around ePROs in nephrology is emerging. Studies from North America demonstrate the feasibility of electronic capture of PROs in HD (34, 35) and an Australian pilot study is currently exploring the feasibility and acceptability of ePROs capture and feedback among patients receiving HD in the Symptom monitoring With Feedback Trial (36). In advanced CKD populations, the RePROM study is piloting the use of an ePRO for remote symptom monitoring in the UK (37), whilst the AmbuFlex telepro system is used to

For peer review only - http://bmjopen.bmj.com/site/about/guidelines.xhtml

manage renal follow up in Denmark (38). Aiyegbusi et al. (2019) explored key stakeholder perspectives on the use of PROs in these pre-dialysis patients and their findings are consistent with this study, particularly regarding potential benefits and administrative aspects (29); suggesting PRO data collection early in the patient pathway can be instituted and continue as illness advances through to renal replacement therapy or even conservative care.

A review and synthesis of evidence is currently being undertaken to investigate how PROs might work to enhance patient-centred care in renal settings, to offer strategy and guidance at individual and aggregate levels of decision-making (39). For effective multiple uses of data, implementation needs to be viewed within the context of complex data linkage and accessibility issues. Studies in the UK and Australia are currently exploring and testing such linkage of symptoms and QoL data to the Australia and New Zealand Dialysis and Transplant Registry and UK Renal Registry respectively (36, 40, 41)

However, the purpose of data collection must be clear. Participants in this study confused the concepts of PROs versus PREMs, understandable given that experience of undertaking HD is intricately linked to both HRQoL and symptoms. The integrated use of both measures, side by side, warrants further investigation. Anxieties about purpose and data sharing could affect patient engagement; clear communication of roles and expectations should be undertaken.

Whilst guidance exists on what outcomes to measure in research (5, 6) and routine practice (9) this has not necessarily been centred on routine capture and feedback to guide individual care. Consequently, it is not clear which measure(s) should be used to capture data in a non-burdensome way, whilst providing sufficient measurement properties to support both single patient monitoring and aggregation of data where required, meaning further enquiry is needed.

For peer review only - http://bmjopen.bmj.com/site/about/guidelines.xhtml

The general lack of awareness and experience of PROs and concern that PROs capture may highlight issues that they either should not or could not deal with, leads to a significant finding that PROs implementation must sit within a broader educational framework. PROs could be used to support wider initiatives and training, especially in the nursing group, who can then cascade information and self-management skills to patients, thereby increasing overall patient health literacy. This could be considered as closing an 'information loop'. There is already evidence that patient education is associated with better patient outcomes (42) and new strategies and quality improvement programs exist such as Shared HD, a UK programme aiming to support patients receiving in-centre haemodialysis to be more independent and confident in participating in aspects of their own care (43). Potential barriers are cost and resources, but increasingly, accredited online learning facilities are available. However, it is acknowledged, staff in this study indicated a preference for face-to-face training. Education and Training on PROs would be needed for all stakeholders.

This study identified time and workflow interruptions as key barriers to implementation. Rotenstein et al. (2017) found that following initiation of routine PROs collection in surgical settings, such concerns shifted as clinicians became comfortable with new processes. There was even a suggestion that PROs could enhance physician satisfaction and prevent 'burnout' (44). Tong et al. (2018) interviewed nephrologists, who also identified resource constraints and uncertainties in how to prioritize, measure, and manage a range of competing comorbidities and broader quality of life outcomes in a clinical setting that is technically demanding and traditionally focused on biochemical factors (45); findings mirrored by this study. Such anxieties will need addressing.

During the SARS-CoV 2 pandemic, data from the UK Renal Registry showed that patients undergoing HD experience a relative risk of mortality of 45.5% compared to the general population (46). This vulnerable population has been unable to effectively shield, requiring

For peer review only - http://bmjopen.bmj.com/site/about/guidelines.xhtml

regular dialysis treatment with associated risks around shared transport and waiting areas. Various strategies have been employed to reduce risks (47) but new ways of care delivery were required. The pandemic has irrevocably changed healthcare, with increased use of virtual services (48) and remote clinic visits. The addition of ePROs to readily available biomedical data means that clinicians could more effectively deliver patient-centred care without the necessity of the patient being physically present. This study also suggests that remote symptom monitoring could offer patients the safety and confidence to dialyse at home and arguably ePROs could assist the HomeHD service in maintaining its patient-centred approach while serving ever increasing numbers.

There are limitations to this study. Participants needed to be English speakers; this could affect transferability of findings. Views of carers were not specifically sought but their influence and importance were clearly identified by patients, suggesting further research targeting this group is warranted. Critically, data collection took place pre-SARS-COV-2 pandemic and experiences and perceptions around digital data capture and new approaches to healthcare delivery may well have shifted.

To conclude, while the nephrology community, both patients and HCPs, are largely supportive of the concept of ePROs, there remain caveats. Stakeholders need to be convinced by empirical evidence, considering the best available measures and methodological considerations. A comprehensive implementation strategy is required to support delivery, and findings from this study can assist, by identifying the priorities and concerns of both patients and clinicians, including a timely understanding of facilitators and barriers.

Author's Contributions:

NA, CMcM, MC, MD, PC, OA, DK: Substantial contribution to the conception and design of the study
NA: Data collection
NA, CMcM, DK Analysis and interpretation of data for the work

For peer review only - http://bmjopen.bmj.com/site/about/guidelines.xhtml

NA, CMcM, MC, MD, PC, OA, DK: Drafting and revising the work critically for important intellectual content **and** final approval of version to be published **and** agreement to be accountable for all aspects of the work in ensuring that questions related to the accuracy or integrity of any part of the work are appropriately investigated and resolved.

Acknowledgments:

PROM-HD PPI Group

Renal Research Patient Advisory Group

Queen Elizabeth Hospital Kidney Patients Association (QEKPA)

For peer review only

For peer review only - http://bmjopen.bmj.com/site/about/guidelines.xhtml

REFERENCES:

1. Bikbov B, Purcell CA, Levey AS, Smith M, Abdoli A, Abebe M, et al. Global, regional, and national burden of chronic kidney disease, 1990–2017: a systematic analysis for the Global Burden of Disease Study 2017. The Lancet. 2020;395(10225):709-33.
2. Almutary H, Bonner, A, Douglas, C. Symptom burden in Chronic Kidney Disease: a review of recent literature. Journal of Renal Care. 2013;39(3):140-50.
3. Abdel-Kader K, Unruh ML, Weisbord SD. Symptom burden, depression, and quality of life in chronic and end-stage kidney disease. Clinical Journal of The American Society of Nephrology: CJASN. 2009;4(6):1057-64.
4. KDIGO. KDIGO 2012 Clinical Practice Guideline for the Evaluation and Management of Chronic Kidney Disease. Kidney International Supplements 2013;3(1).
5. Evangelidis N, Tong A, Manns B, Hemmelgarn B, Wheeler DC, Tugwell P, et al. Developing a Set of Core Outcomes for Trials in Hemodialysis: An International Delphi Survey. Am J Kidney Dis. 2017.70 (4) Pages 564-575
6. Tong A, Manns B, Hemmelgarn B, Wheeler DC, Evangelidis N, Tugwell P, et al. Establishing Core Outcome Domains in Hemodialysis: Report of the Standardized Outcomes in Nephrology-Hemodialysis (SONG-HD) Consensus Workshop. Am J Kidney Dis. 2017;69(1):97-107.
7. Viecelli AK, Tong A, O'Lone E, Ju A, Hanson CS, Sautenet B, et al. Report of the Standardized Outcomes in Nephrology-Hemodialysis (SONG-HD) Consensus Workshop on Establishing a Core Outcome Measure for Hemodialysis Vascular Access. Am J Kidney Dis. 2018.71 (5) Pages 690-700
8. Urquhart-Secord R, Craig JC, Hemmelgarn B, Tam-Tham H, Manns B, Howell M, et al. Patient and Caregiver Priorities for Outcomes in Hemodialysis: An International Nominal Group Technique Study. Am J Kidney Dis. 2016;68(3):444-54.
9. Verberne WR, Das-Gupta Z, Allegretti AS, Bart HAJ, van Biesen W, Garcia-Garcia G, et al. Development of an International Standard Set of Value-Based Outcome Measures for Patients With Chronic Kidney Disease: A Report of the International Consortium for Health Outcomes Measurement (ICHOM) CKD Working Group. Am J Kidney Dis. 2019;73(3):372-84.
10. International Consortium for Health Outcomes Measurement (ICHOM). Chronic Kidney Disease: Data Collection Reference Guide. ICHOM; 2017 Oct 25th 2017. Contract No.: Version 1.0.https://www.ichom.org/wp-content/uploads/2020/02/ICHOM-Chronic-Kidney-Disease-Reference-Guide-20171025-1.00.pdf Last Accessed 20.01.2021
11. FDA. Guidance for industry. Patient reported outcomes measaures: Use in medical product development to support labeling claims. 2009. Docket No:FDA-2006-D-0362. https://www.fda.gov/media/77832/download Last Accessed 20.01.2021
12. Calvert M, Kyte D, Price G, Valderas JM, Hjollund NH. Maximising the impact of patient reported outcome assessment for patients and society. BMJ. 2019;364:k5267.
13. Basch E, Deal AM, Dueck AC, Scher HI, Kris MG, Hudis C, et al. Overall Survival Results of a Trial Assessing Patient-Reported Outcomes for Symptom Monitoring During Routine Cancer Treatment. JAMA. 2017;318(2):197-8.
14. Gair R, M, Stannard, C, Wong, E, Hawkins, J, Van der Veer, S, N, Farrington, K, Hope, J, Fluck, R. Transforming-Participation-in-Chronic-Kidney-Disease - Programme Report. 2019.https://www.thinkkidneys.nhs.uk/ckd/wp-content/uploads/sites/4/2019/01/Transforming-Participation-in-chronic-kidney-disease-1.pdf Last Accessed 20.01.2021
15. Anderson NE, Calvert M, Cockwell P, Dutton M, Kyte D. The Use of Patient-Reported Outcomes in Patients Treated With Maintenance Hemodialysis: A Perspective. Am J Kidney Dis. 2019.74 (3) Pages399-406
16. Anderson NE, Calvert M, Cockwell P, Dutton M, Aiyegbusi OL, Kyte D. Using patient-reported outcome measures (PROMs) to promote quality of care in the management of patients with established kidney disease requiring treatment with haemodialysis in the UK (PROM-HD): a qualitative study protocol. BMJ Open. 2018;8(10):e021532.

For peer review only - http://bmjopen.bmj.com/site/about/guidelines.xhtml

17. Etikan I. Comparison of Convenience Sampling and Purposive Sampling. American Journal of Theoretical and Applied Statistics. 2016;5(1).Pages1-4
18. Standardised Outcomes in Nephrology (SONG) . The SONG Handbook. Version 1. Sydney, Australia 2017. Available from: songinitiative.org/reports-and-publications/.Last accessed 20.01.2021
19. Hays R D KJD, Mapes D L, Coons S J, Amin N, Carter W B, Kamberg C. Kidney Disease Quality of Life Short Form (KDQOL-SF): A Manual for use and scoring. Version 1.3 In: RAND, editor. 1997.https://www.rand.org/content/dam/rand/pubs/papers/2006/P7994.pdf Last Accessed 20.01.2021
20. Hays RD, Kallich JD, Mapes DL, Coons SJ, Carter WB. Development of the kidney disease quality of life (KDQOL) instrument. Quality of Life Research. 1994;3(5):329-38.
21. Scale PPCO. POS Palliative Care Outcome Scale 2017 [Available from: https://pos-pal.org/maix/ipos-renal-in-english.php. Last Accessed 20.01.2021
22. Braun V, Clarke V. Can I use TA? Should I use TA? Should I not use TA? Comparing reflexive thematic analysis and other pattern-based qualitative analytic approaches. Counselling & Psychotherapy Research.18 Oct 2020. https://doi.org/10.1002/capr.12360
23. Damschroder LJ, Aron DC, Keith RE, Kirsh SR, Alexander JA, Lowery JC. Fostering implementation of health services research findings into practice: a consolidated framework for advancing implementation science. Implement Sci. 2009;4:50.
24. van Oers HA, Teela L, Schepers SA, Grootenhuis MA, Haverman L, PROMs I, et al. A retrospective assessment of the KLIK PROM portal implementation using the Consolidated Framework for Implementation Research (CFIR). Qual Life Res. 2020.https://doi.org/10.1007/s11136-020-02586-3
25. Kerr CN, A. Wild, D. Assessing and demonstrating data saturation in qualitative inquiry supporting patient-reported outcomes research. Expert Review of Pharmacoeconomics & Outcomes Research. 2010;10(3):269-81.
26. MARY E. CHARLSON PP, KATHY L. ALES and C. RONALD MACKENZIE A NEW METHOD OF CLASSIFYING PROGNOSTIC COMORBIDITY IN LONGITUDINAL STUDIES: DEVELOPMENT AND VALIDATION J Chron Dis 1987;40(5):373-83.
27. UK Renal Registry. UK Renal Registry 22nd Annual Report – data to 31/12/2018,. Bristol, United Kingdom; 2020.https://renal.org/audit-research/22nd-annual-report-data-31122018
28. Coulter A. Measuring what matters to patients. BMJ. 2017;356:j816.
29. Aiyegbusi OL, Kyte D, Cockwell P, Marshall T, Dutton M, Walmsley-Allen N, et al. Patient and Clinician Perspectives on Electronic Patient-Reported Outcome Measures in the Management of Advanced CKD: A Qualitative Study. Am J Kidney Dis. 2019.74 (2) Pages 167-178
30. Tong A, Winkelmayer WC, Wheeler DC, van Biesen W, Tugwell P, Manns B, et al. Nephrologists' Perspectives on Defining and Applying Patient-Centered Outcomes in Hemodialysis. Clin J Am Soc Nephrol. 2017;12(3):454-66.
31. Denis F, Basch EM, Lethrosne C, Pourel N, Molinier O, Pointreau Y, et al. Randomized trial comparing a web-mediated follow-up via patient-reported outcomes (PRO) vs. routine surveillance in lung cancer patients: Final results. Journal of Clinical Oncology. 2018;36(15_suppl):6500-.
32. Denis F, Basch, E, Septans, A, Bennouna, J, Urban,T, Dueck, A, Letellier, C. Two year survivial comparing web-based symptom monitiring vs routine surveillance following treatment for lung cancer. JAMA. 2019;321(3):306-7.
33. Greenhalgh J AP, McCluskey S, Takeuchi E, Velikova G. How do doctors refer to Patient Reported Outcome Measures (PROMs) in oncology consultations. Quality of Life Research. 2013;22(5):939-50.

For peer review only - http://bmjopen.bmj.com/site/about/guidelines.xhtml

34. Schick-Makaroff K, Molzahn A. Strategies to use tablet computers for collection of electronic patient-reported outcomes. Health & Quality of Life Outcomes. 2015;13:2.https://doi.org/10.1186/s12955-014-0205-1
35. Gabbard J, McLouth CJ, Brenes G, Claudel S, Ongchuan S, Burkart J, et al. Rapid Electronic Capturing of Patient-Reported Outcome Measures in Older Adults With End-Stage Renal Disease: A Feasibility Study. The American journal of hospice & palliative care. 2020:.https://doi.org/10.1177/1049909120954805
36. Viecelli A, Dansie K, McDonald S, Jesudason S, Duncanson E, Bennett P, et al. Symptom monitoring with feedback trial (SWIFT) pilot to explore the feasibility and acceptability of electronic patient reported outcome measures (e-proms) data capture and feedback. Nephrology Dialysis Transplantation. 2020;35 (SUPPL 3):iii137.
37. Kyte D, Bishop J, Brettell E, Calvert M, Cockwell P, Dutton M, et al. Use of an electronic patient-reported outcome measure in the management of patients with advanced chronic kidney disease: the RePROM pilot trial protocol. BMJ Open. 2018;8(10):e026080.
38. Schougaard L M LLP, Jessen A et al. AmbuFlex: tele-patient-reported outcomes (telePRO) as the basis for follow up in chronic and malignant diseases. Qual Life Res. 2016;25(3):525-34.
39. Schick-Makaroff K, Thummapol O, Thompson S, Flynn R, Karimi-Dehkordi M, Klarenbach S, et al. Strategies for incorporating patient-reported outcomes in the care of people with chronic kidney disease (PRO kidney): a protocol for a realist synthesis. Syst Rev. 2019;8(1):20.
40. Morton RL, Lioufas N, Dansie K, Palmer S, Jose M, Raj R, et al. Patient reported outcome measures in australian and new zealand renal units: An anzdata survey of current practice. Nephrology.23:32.2019 https://doi.org/10.1111/nep13577
41. Van Der Veer SN, Ercia A, Caskey FJ, Farrington K, Jury F, Rees M, et al. Developing an Intervention to Implement Electronic Patient-Reported Outcomes in Renal Services in the UK. Studies in Health Technology & Informatics. 2020;270:936-40.
42. Narva AS, Norton JM, Boulware LE. Educating Patients about CKD: The Path to Self-Management and Patient-Centered Care. Clin J Am Soc Nephrol. 2016;11(4):694-703.
43. Wilkie M, Barnes T. Shared Hemodialysis Care: Increasing Patient Involvement in Center-Based Dialysis. Clin J Am Soc Nephrol. 2019;14(9):1402-4.
44. Rotenstein LS, Huckman RS, Wagle NW. Making Patients and Doctors Happier — The Potential of Patient-Reported Outcomes. New England Journal of Medicine. 2017;377(14):1309-12.
45. Tong A, Winkelmayer WC, Wheeler DC, Biesen WV, Tugwell P, Manns B, et al. Nephrologists' perspectives on defining and applying patient-centered outcomes in hemodialysis. Clinical Journal of the American Society of Nephrology. 2017;12(3):454-66.
46. Savino M, Casula A, Santhakumaran S, Pitcher D, Wong E, Magadi W, et al. Sociodemographic features and mortality of individuals on haemodialysis treatment who test positive for SARS-CoV-2: A UK Renal Registry data analysis. PLoS One. 2020;15(10):e0241263.
47. Roper T, Kumar N, Lewis-Morris T, Moxham V, Kassimatis T, Game D, et al. Delivering Dialysis During the COVID-19 Outbreak: Strategies and Outcomes. Kidney Int Rep. 2020;5(7):1090-4.
48. Hull SA, Rajabzadeh V, Thomas N, Hoong S, Dreyer G, Rainey H, et al. Do virtual renal clinics improve access to kidney care? A preliminary impact evaluation of a virtual clinic in East London. BMC Nephrol. 2020;21(1):10.

For peer review only - http://bmjopen.bmj.com/site/about/guidelines.xhtml

Box 1 Key Findings: summary of direct quotes

Key Finding	Illustrative Quotation
**1. Intervention Characteristics**	
**The importance of demonstrating efficacy/benefit**	*'one is convincing those patients and doctors and nurses that it's actually of use to man or beast. I think that is going to be the main barrier. HCP11.*<br>*'I think if you could then see that it changed a consultation that meant that you went, 'Oh, I didn't know you were having that issue; I can do something about that'. The difficulty is if they then, it drives a lot of conversations that, you know, saying, 'Yeah, can't really do anything about then', and then everybody may well think well this hasn't changed anything so, you know, is it making you feel better writing it down? Don't know really. So if there's not an outcome difference to it then it just becomes a kind of form-filling exercise but there's not obviously a benefit'. HCP16*<br><br>*'If they haven't got a point for collecting them that isn't going to be actioned or put into a specific piece of research, then they shouldn't be there……'Yes, it makes you unwilling to invest your own time in something that you hadn't seen any benefit from in the past….If it's just a box ticking exercise for your unit or whatever, to say that I've complied with some directive and nothing is going to change, then why would you.' Patient JI18*
**The intervention must be flexible/offer choices**	*'I guess we have to give the option to the patient whether and how they want to fill this in or not because some patients may not want to fill it in. And I can think of a few patients on my dialysis unit that probably would not want to fill this in.'HCP14*
**Practical considerations**	See Box 2 for key findings and illustrative direct quotes
**2. Outer Setting**	
**Identifying and measuring Outcomes that matter**	*I need to go out. I need to keep this going. I need to keep feeling that I'm worth something by bringing home a wage and working' Patient YZ13*<br><br>*IV: Yes, definitely. We see that all the time don't we? I mean you know, we see that all the time. I mean*

For peer review only - http://bmjopen.bmj.com/site/about/guidelines.xhtml

How PRO's fit into the 'Bigger Picture'	*that's the battle, to get people to dialyse three times a week rather than twice a week [yeah, yeah]. So you know, I'm having this battle with a man in XUnit at the moment who's decided to go and dialyse twice a week and he tried it for a few weeks. And he said, 'Oh, I feel perfectly well. So I'm just going to carry on dialysing twice a week' and I said, 'Well, that's not the point' [laughs][absolutely]. And he said, 'Yeah, but I feel fine. I get more time, I get more spare time'. HCP13*<br>*'It does really knock the quality of life, some people tend to be more worried about the quantity of life but I'm interested in the quality of life because not having quality of life, I can understand why some people do commit suicide, there's no quality' Patient ST10*<br><br>Continuity of care and communication with Primary Care:<br>*' Yeah, the other thing I find with my GP for example, now she rang me, I have monthly blood test here and every 12 months you go to the GP and I go to the diabetic clinic, my diabetes is good…….so the doctor (GP) rang up and said, I think you're anaemic, I said well I've had these blood tests at the dialysis centre and nobody phoned up that I was anaemic……'Patient ST10*
**3. Inner Setting**	
**Compatibility of PROs and approaches to delivery of care in HD settings**	Task centred care versus patient centred care:<br>*'we don't always see the bigger picture, we're so focused on getting that needle in to get dialysis, that we forget, oh my god, there's a person on the end of that arm…..' HCP12*<br><br>*'in-centre, it's looking at the patient, home haemo it's looking at the person' HCP08*<br><br>*'I think the nurses have got time to get to know the patients to a certain extent as far as medically, their medication is involved. You know, how poorly they are, obviously what they need. Unfortunately, I don't think they have time to carry that a bit further…..' HCP02*
**PRO's as part of a wider educational framework – 'the information loop'**	Implementation must be part of a wider framework of training, education, and support:<br>*' this will be exciting if implemented correctly, but there's a whole massive amount of work, it's not just this, it's everything else around it yeah, I think so' HCP17*<br><br>Staff Education requirements:<br><br>*'So I guess education would be vital, but they're about communication skills, they're about learning what matters in chronic disease management. And it would also be about, because that's burdensome, it's hugely draining to do it day in day out with a similar group of people. And you would need to put in support, raising the education*

For peer review only - http://bmjopen.bmj.com/site/about/guidelines.xhtml

	for them is one, raising awareness. But I also think if you are hearing people's symptoms, which are as I say some of them you can't resolve and you're hearing them day in day out, you need somewhere to offload to as well. So it's like, but building a good structure around what's needed. But that doesn't mean don't talk about them.' HCP05<br><br>Health Literacy of patients:<br>*Talking about monthly QA reports – ' ….and we all get these, telling us about our potassium, and somebody says to me, do you understand them? I say, I haven't got a clue, he says nor me, I says, all I know, when I go inp there, they tell me that's okay, that's okay, that's okay, I said but I haven't got a clue' Patient BA14*
**4. Characteristics of Individuals**	
Previous experience of using PROs	*'I mean I've used them in various research projects………And that's one of the reasons why I haven't routinely used them in clinical practice because I think they're fine for research, but I haven't found one that's hugely clinically, that I feel I'd really want to use.' HCP06*
Concept of PROs versus PREMs	*'IV:[PROMs & PREMs] I get confused and I think there's crossover and I find it quite difficult to draw a line between experience and outcome. Because for example, the experience of a patient. So, tiredness for example can be strongly affected by a patient experience. So, you have travelled six hours to get there or waited for your transport, your experience, it affects your tiredness which is – I think – I'm not sure always it's a useful distinction' HCP12*<br><br>*'I: Yeah. So do you think we should be measuring them (PROMs and PREMs) at the same time?*<br>*IV: I think that they should be part of the same thing. I think that they should be seen so that we can pick apart whether people are happy with the service that they're getting but also how that interplays with their health and their feelings and their symptoms so I think the two things overlap.' HCP16*
Multidimensionality of ESKD and consequent challenges	*'…..it's most of the time that I get anxious with social situations because of my other medical problems. It's not just about my kidney problems and then dealing with the social aspect. It's my kidney problems, plus my other medical problems and then the social situation. I have to deal with my other medical problems before I can even contemplate going out with family or friends…..or shopping and things like that' Patient WX12*<br><br>*' The main problem? [with dialysis] Social life. Absolutely. It's not just my social life; it's the wife's social life as*

For peer review only - http://bmjopen.bmj.com/site/about/guidelines.xhtml

	well. She's like a nurse to me really. Without wife, I don't know where I'd be. I've got two lads but you can't rely on them to be here' YZ13<br><br>*'If we can't do much about it, then it's not our problem, sort of thing. That's one of those barriers, I think there's the worry that it uncovers things that are uncomfortable to talk about….' HCP15*<br><br>Acknowledgement that there are aspects of care that do not fall within doctors 'remit' *'things that aren't necessarily medical, that may relate to social services or other things that are driving a symptom or feeling that relates more to social care, to a dietitian or other members of the team….' HCP16*
**5. Process of implementation**	
Supporting the process of implementation<br><br><br><br><br><br><br><br><br><br>Barriers to effective implementation: Time/attitude<br><br><br><br><br><br><br><br>Facilitators to effective implementation: How PROs might deliver a relative advantage over current systems of care	Importance of leadership and champions:<br>*'Time, resources, you know, if someone's not here, say, if it was me implementing it, if I'm not here, I'm thinking, will it get done? It's as simple as that' HCP17*<br><br>*'You might need to bring somebody else in from outside, yeah but that runs its own complications so what's the answer, a patient representative might be an answer, somebody who knows dialysis.' HCP17*<br><br>Bottom up approach:<br>*'Often there are new things that are introduced and when you've just got to do it attitude. Now that just gets peoples backs up to start with………have a proper process in place to prepare people' HCP07*<br><br>*'They [Doctors] haven't got time anymore. We all know that, but it's nice to not be rushed along and ignored' Patient MN20*<br><br>*' It does annoy me when we have problems with logging in and passwords' Patient WX12*<br><br>*'So it has to be easy access for the patient. It's pointless having something they can't actually get to and that's scary or frustrating to get onto, to log on or something, yeah.' HCP05*<br><br>Could reduce workload:<br>*'I find it really interesting how people would say that this is an increase in workload, when actually this is things that you need to be looking at anyway. For me this is nothing out of what I'm already looking at in every patient*

For peer review only - http://bmjopen.bmj.com/site/about/guidelines.xhtml

delivery	*and of course it will be an increase in workload if you don't look at them.' HCP17*<br><br>Potentially time saving:<br>*I think the PROMs, certainly one is useful when they fill them in but quite often they haven't filled them in and it's then sometimes difficult to ascertain have they not filled them in because they have got symptoms but they just aren't necessarily engaging or thinking oh therefore I will fill this in or whether they haven't filled it in 'cause they're fine. But then at the same time it's quite easy to ask a patient and say, 'Have you not filled this in?', because actually they're fine and then they'll say, 'Yes, I'm fine. There's nothing to worry about', sort of thing and then in a way you can think well I can, I don't necessarily need to give you targeted questions 'cause you've already got the opportunity to do that. Sometimes it then speeds things up. HCP06*

For peer review only - http://bmjopen.bmj.com/site/about/guidelines.xhtml

Box 2 -Practical Considerations and illustrative direct quotes

**Practical Considerations**	**Key Findings**	
	**Patient Participants**	**HCP Participants**
**Frequency (to allow for accurate recall)**	Wide variation of responses from 1 week to 12 months. Agreement that it needed to match recall whilst allowing for treatment responses. Several patients would complete as frequently as asked – demonstrating no preference.	Wide variation of responses from, as required to 12 months. Focus on practicality i.e. how often they thought patients would complete and time burden rather than sensitivity to changes and recall. Before routine clinic visit was popular response.
**Timing (in relation to dialysis cycle)**	Some patients wanted to complete during dialysis but noted this could be practically difficult, particularly if their access was in dominant arm. Most did not want to complete immediately after dialysis, the majority requiring some recovery time.	*'Horses for courses' HCP11*<br>Options, in-centre before or during session or patients choice at home (need time frame for completion? i.e. within 5 day window)<br>General agreement that straight after dialysis patients are very fatigued, so not optimum time for completion.<br>Effects of cognitive status on concentration levels – ability to partially complete and come back later, recall bias will be relevant. Need to consider are you asking about today, last 2 weeks, last month, when designing tool.
**Setting of completion (Home/in-centre)**	Some favoured completion in centre, others preferred to complete at home, particularly if carer assistance required.	Patients needed options (home/Unit) – some patients need assistance so carers could help (unlikely nurses would have time)<br>It was suggested better returns if encouraged to complete at unit: at home patients are 'distracted by life'.<br>*Will responses be different if completing at home – does that matter? 'either way, you're gonna get the reality of their life, so perhaps it doesn't matter' HCP05*<br>Lack of privacy in-centre noted by number of respondents
**Mode of capture**	Varied responses – digital, paper or no preference. Proponents of digital capture displayed no obvious preference for choice of device. Only one patient had no access to a digital device at home.	Advantages of electronic collection raised (real time review, less admin, less likely to lose returns, easier to track responses, to review responses/feedback) but option of paper needs to be available, as some patients would be unable to complete digitally, and who would provide this assistance? Some pondered that current digital resources i.e. PatientView are not widely

For peer review only - http://bmjopen.bmj.com/site/about/guidelines.xhtml

	Preference for paper often reflected lack of confidence in ability to complete digitally, a number said they would need assistance to complete electronically<br>*'IV: I feel like my confidence is down and that needs to build back up again before I use the computer.' Patient CD02*	used. IT systems require resource for infrastructure, upkeep and training.
**Readability**	No jargon or medical terms – got to be easy to read and understand.<br>Guidance around more complex constructs<br>Availability in of measure in other languages<br>*' you may find you need to print them in their own Urdu or whatever, so that they do understand them. And that has to be very careful that the question doesn't get lost in the translation' Patient MN07*	Simple and clear rating scales for staff undertaking interpretation.<br>Need to be culturally sensitive/valid and available in languages other than English.
**Length of questionnaire**	Generally, length was not an issue, provided patients could see a purpose to completion. There was a dislike of repetitive questions. Potential time burden of lengthy questionnaires, seen as an inconvenience for carers<br>*'So the other question I was going to ask was were you put off by the length of some of those questionnaires?'*<br>*IV: No I wasn't, no, you know I'd put aside the time to do it so no I wasn't, I could see the point of them.' UV11*	Consensus that the shorter the questionnaire, the better.<br>Some commented that current validated PROMs were much too lengthy.
**Form of Feedback**	No overall preference: could be letter, email, or discussion. Most happy to use graphical data. However, important that timely feedback given and PROMs completion *'not just a tick box exercise'.* Some patients wanted carers involved in feedback process.<br>*'Because I don't do emails and sometimes my eyesight, I can't able to read. Yeah. I prefer in person'. Patient GHO4*<br><br>The need for timely feedback was made:<br>*I: So you'd want to see some sort of feedback within two weeks.*<br>*IV: Two weeks and they sort it out. Not leave it for months*	Consensus that electronic feedback is ideal. Responses outlined in letter to patient or face to face in clinic to allow 'probing' – but this requires resource.<br>Agreement that longitudinal data would be advantageous to see trends – preference for graphical rather than tabular form – but scales need to be well thought through, 0-3 not considered 'sensitive' enough to be able to show changes – preference for EQ-5D thermometer scale… *'but a 'Blunt tool'- good clinician should pick up sensitive changes before PROMs would' HCP11*<br>No time to read lots of free text

For peer review only - http://bmjopen.bmj.com/site/about/guidelines.xhtml

	and months and years and years, no good at all. Patient KL06<br><br>*'As well as giving helpful information to help you manage your condition, it would also be useful to have information about what causes certain things in order to put your mind at rest, if you know what I mean?' Patient CD02*	
**Setting of feedback**	Currently most consultations take place while the patient is dialysing. Patients gave mixed responses to the wish to be seen in private, most did not want additional or extended visits at the dialysis unit and seemed resigned to being seen in open bays.	If medical staff were providing feedback in person, pragmatically best to do this in clinic, acknowledged this can take place at the bedside during dialysis and this may affect
**Who provides feedback**	No overall preference, but one patient felt more comfortable talking to nursing staff.	Variation on responses – some thought medical staff (but concerns raised around time and workload) other thought nurses (some anxiety regarding time constraints and knowledge/experience of junior nursing staff to know how to manage symptoms or issues raised by PROMs)<br>*Need clear process for review and feedback – 'can't have 6 nurses going back to patient to discuss responses HCP16'*.

For peer review only

Table 1: Patient Participant Characteristics

Variable	Count
**Age**	
Age under 40	2/22 (9%)
40-49	3/22 (13%)
50-59	5/22 (23%)
60-69	3/22 (14%)
70-79	6/22 (27%)
Over 80	3/22 (13%)
**Gender**	
Male	12/22 (55%)
Female	10/22 (45%)
**Ethnicity**	
White -British	16/22 (73%)
Black- British	4/22 (18%)
Asian/Asian British	2/22 (9%)
**In centre Dialysis**	15/22 (68%)
Morning sessions	12/15 (80%)
Home HD	7/22 (32%)
**Current Vascular Access**	
Arterio-Venous Fistula	19/22 (86%)
Arterio-Venous Graft	0/22 (0%)
Central Venous Catheter	3/22 (14%)
**Charlson Comorbidity Score**	
<2	1 (4%)
3-4	5 (23%)
5-6	4 (18%)
7-8	6 (27%)
9-10	5 (23%)
11-12	1(4%)
13+	0 (0%)
**Time since dialysis commencement (years)**	
≤ 5	11/22 (50%)
6-10	5/22 (23%)
11-15	3/22 (14%)
16-20	1/22 (4.5%)
>20	2/22 (9%)
Note n=22	

Charlson Comorbidity Index (CCI) (26) quantifies an individual's burden of disease and corresponding 1-year mortality risk. The index adjusts for 17 comorbidities, each one classified with a validated score of 1–6 points, based on the adjusted relative risk of 1-year mortality. The final total score is used to calculate the probability of survival. The index is being used in this context to demonstrate the overall disease burden of the population under study.

For peer review only

Table 2: Health Care Professional Participant Characteristics

Variable	Count
**Gender**	
Male	5/17 (29%)
Female	12/17 (71%)
**Ethnicity (Office of National Statistics Categories)**	
White -British	9/17 (53%)
Other white background	2/17 (12%)
Black- British	1/17 (6%)
Asian/Asian British	1/17 (6%)
Any other Asian background	2/17 (12%)
Black African	1/17 (6%)
White and Black Caribbean	1/17 (6%)
**Role**	
Consultant Nephrologist	5/17 (29%)
Consultant Surgeon	1/17 (6%)
Registered Nurse	9/17 (53%)
Non-registered Healthcare Assistant	1/17 (6%)
Administrative Assistant	1/17 (6%)
**Time working across HD setting (years)**	
≤ 5	1/17 (6%)
6-10	4/17 (24%)
11-15	3/17 (18%)
16-20	2/17 (12%)
>20	7/17 (41%)
Note n=17	

For peer review only

For peer review only - http://bmjopen.bmj.com/site/about/guidelines.xhtml

For peer review only

For peer review only - http://bmjopen.bmj.com/site/about/guidelines.xhtml

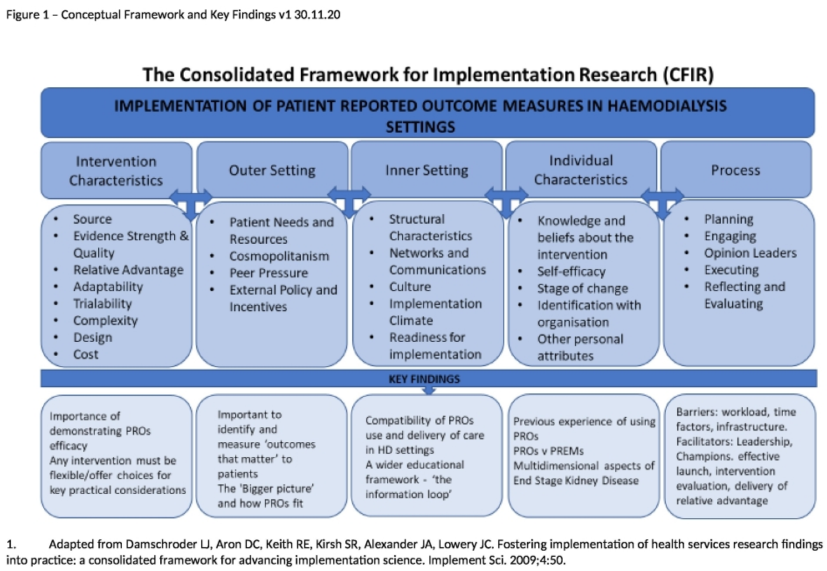

Figure 1 Conceptual Framework

1237x874mm (72 x 72 DPI)

Supp Table 1 Patient saturation table and interview characteristics

	Value
**Patient Interviews**	
Number invited/Number participating	22/41
Reasons for non-participation	Did not meet eligibility criteria, did not want to participate (transport issues, did not want to do research)
Duration of patient interviews (min)	Range 24-84<br>Mean 50 mins
**Patient Saturation Data**	
No of codes generated	110
Point of data saturation	15th interview
Note n=17	

		1	2	3	4	5	6	7	8	9	10	11	12	13	14	15	16	17	18	19	20	21	22
**1 Innovation Characteristics**																							
1A Innovation Source													x						x				
1B Evidence strength and quality		/	/	/	/	/	/	/	/	/	/	/	/	/	/	/	/	/	/	/	/	/	/
1C Relative Advantage	Perception of advantage	x	x		x	x	x	x			x	x	x		x	x	x		x		x	x	
	Intractable problems								x			x		x	x	x					x		
	Potential to cause anxiety										x			x	x		x			x	x	x	
1D Adaptability	Adaptability of intervention												x						x				
	Use within research	x	x	x	x			x	x			x	x	x		x	x		x				
	Importance of choices in how and what is answered	x	x	x				x					x				x	x				x	
1E Trialability		/	/	/	/	/	/	/	/	/	/	/	/	/	/	/	/	/	/	/	/	/	/
IF Complexity								x											x				
IG Design and Packaging	Frequency	x	x		x		x	x		x	x	x		x	x	x	x			x		x	x
	Length	x	x	x	x		x	x		x	x	x	x	x	x	x	x	x	x	x	x		x
	Language/Readability					x		x	x	x	x	x	x		x		x	x			x		x
	Mode	x	x	x	x	x	x	x	x	x	x	x		x	x	x	x	x	x	x	x	x	x

For peer review only

Supp Table 1 Patient saturation table and interview characteristics

		1	2	3	4	5	6	7	8	9	10	11	12	13	14	15	16	17	18	19	20	21	22
	Setting	x		x	x	x		x	x		x	x	x	x		x	x	x		x	x	x	x
	Timing	x	x		x	x	x		x	x	x			x	x	x		x	x	x		x	x
	Outcomes that matter	x	x		x	x	x			x			x	x	x	x			x	x		x	
	Current Measures				x	x	x	x	x		x	x	x	x	x		x	x	x		x	x	x
	Safety Alerts	x																	x			x	
	Self-help information		x						x				x	x			x					x	
	ComputerAdaptiveTechnology										x	x		x		x	x		x	x			
	Review and Feedback	x	x		x		x	x	x	x	/	x	x	/	x	x	/	x	x	x		x	x
	Sensitivity to change	/	/	/	/	/	/	/	/	/		/	/		/	/		/	/	/	/	/	/
IH Cost																			x				
**2 Outer Setting**																							
2A Needs and resources	Identification		x	x	x		x	x	x			x	x	x	x			x	x	x	x	x	x
	Barriers	x	x	x	x	x	x	x	x	x	x	x	x	x	x	x	x	x	x	x	x	x	x
	Facilitators	x	x	x	x		x			x	x	x	x	x				x	x	x		x	
2B Cosmopolitanism				x				x			x				x					x			
2C Peer Pressure		/	/	/	/	/	/	/	/	/	/	/	/	/	/	/	/	/	/	/	/	/	/
2D External policies and incentives		/	/	/	/	/	/	/	/	/	/	/	/	/	/	/	/	/	/	/	/	/	/
**3 Inner Setting**																							
3A Structural Characteristics																x							
3B Networks and Communications	Nature and Quality		x	x	x	x	x	x	x	x	x	x	x		x	x		x	x	x	x	x	x
	Shared Decision Making	x	x	x							x	x	x	x	x	x		x		x	x		x
3C Culture											x			x	x	x					x		
3D Implementation Climate	Tension for change		x	x					x			x	x	x	x			x	x	x	x		x
	Compatibility							x				x	x									x	x
	Relative Priority	x			x						x										x	x	
	Organisational Incentives	/	/	/	/	/	/	/	/	/	/	/	/	/	/	/	/	/	/	/	/	/	/

For peer review only

Supp Table 1 Patient saturation table and interview characteristics

		1	2	3	4	5	6	7	8	9	10	11	12	13	14	15	16	17	18	19	20	21	22
	Goals and Feedback	/	/	/	/	/	/	/	/	/	/	/	/	/	/	/	/	/	/	/	/	/	/
	Learning Climate												x										
3E Readiness for Implementation	Leadership Engagement	/	/	/	/	/	/	/	/	/	/	/	/	/	/	/	/	/	/	/	/	/	/
	Available Resources		x		x											x		x				x	
	Access to knowledge and info		x			x	x						x		x	x	x				x	x	x
**4 Characteristics of Individuals**																							
Knowledge and beliefs		x	x	x	x			x	x	x		x		x	x	x	x	x	x	x	x	x	
Self-efficacy	Ability to self-complete	x	x	x		x	x	x	x	x	x	x	x	x	x	x	x	x		x		x	
	Patient as an Expert			x				x				x	x	x	x					x			
	PROMs v PREMs		x		x	x		x											x		x		
Individual stage of change	Previous experience of PROMs usage	x			x	x	x		x	x	x	x	x	x	x	x	x	x	x	x	x	x	x
Individual identification with organisation		x	x		x	x	x	x	x	x	x	x	x	x	x		x			x	x	x	x
Other personal attributes	Multidimensionality of ESKD:																						
	Symptomology	x	x	x	x	x	x	x	x	x	x	x	x	x	x	x	x	x	x	x	x	x	x
	Life on Dialysis	x	x	x	x	x	x	x	x	x	x	x	x	x	x	x	x	x	x	x	x	x	x
**5 Process**																							
5A Planning		/	/	/	/	/	/	/	/	/	/	/	/	/	/	/	/	/	/	/	/	/	/
5B Engaging	Opinion Leaders	/	/	/	/	/	/	/	/	/	/	/	/	/	/	/	/	/	/	/	/	/	/
	Formally appointed leaders					x		x															
	Champions					x							x	x									
	External Change Agents	/	/	/	/	/		/	/	/	/	/	/	/	/	/	/	/	/	/	/	/	/
	Key Stakeholders	/	/	/	/	/		/	/	/	/	/	/	/	/	/	/	/	/	/	/	/	/
	Innovation Participants				x								x										
	Role of Carers	x		x	x		x	x		x			x				x						
5C Executing		/	/	/	/	/	/	/	/	/	/	/	/	/	/	/	/	/	/	/	/	/	/
5D Reflecting and Evaluating		/	/	/	/	/	/	/	/	/	/	/	/	/	/	/	/	/	/	/	/	/	/

Supp Table 1 Patient saturation table and interview characteristics

First time data coded to node in NVIVO	x
Nothing coded to node in NVIVO	/
Nothing coded to node in NVIVO	/

For peer review only

Suppl Table 2 Healthcare Professional Participants: Saturation table and interview characteristics

	Value
**HCP Interviews**	
Number invited/Number participating	17/19
Reasons for non-participation	Competing clinical commitments
Duration of patient interviews (min)	Range 29-63<br>Mean 48 mins
**Patient Saturation Data**	
No of codes generated	134
Point of data saturation	16th Interview
Note n=17	

**Participants n=17**		**1**	**2**	**3**	**4**	**5**	**6**	**7**	**8**	**9**	**10**	**11**	**12**	**13**	**14**	**15**	**16**	**17**
**1 Innovation Characteristics**																		
1A Innovation Source						x										x		
1B Evidence strength and quality		x				x				x		x		x	x	x	x	x
1C Relative Advantage		x	x	x	x	x	x		x	x	x	x	x	x	x	x	x	x
												x	x		x		x	x
	How PROMs might work to deliver advantage		x		x	x	x	x	x	x								
1D Adaptability		x				x												x
	Use within research				x	x							x		x	x	x	
1E Trialability																	x	
IF Complexity		x				x	x		x		x						x	
IG Design and Packaging		x	x	x	x	x	x	x	x	x	x	x	x	x	x	x	x	x
	Frequency	x	x	x	x	x	x	x	x	x	x	x	x	x	x	x		x
	Length			x	x	x	x		x		x	x	x	x	x		x	
	Mode	x	x		x	x			x	x	x	x	x	x		x	x	

For peer review only

Suppl Table 2 Healthcare Professional Participants: Saturation table and interview characteristics

**Participants n=17**		**1**	**2**	**3**	**4**	**5**	**6**	**7**	**8**	**9**	**10**	**11**	**12**	**13**	**14**	**15**	**16**	**17**
	Setting		x	x	x	x		x	x	x	x			x			x	x
	Timing		x	x	x		x		x	x	x	x	x	x	x		x	
	Self-completion		x	x	x	x	x	x				x	x	x	x	x		x
	Outcomes that matter	x	x	x	x		x		x	x	x		x			x		x
	Safety Alerts			x			x				x			x	x	x	x	
	Sensitivity						x		x		x	x						
	Computer Adaptive Technology					x		x	x	x	x	x	x	x		x	x	
	Review and Feedback	x	x	x	x	x	x	x				x	x	x	x	x	x	x
IH Cost														x		x		
**2 Outer Setting**																		
2A Needs and resources						x	x	x		x	x	x	x	x		x		
	Barriers		x		x	x	x	x	x	x	x	x	x	x	x	x	x	x
	Facilitators			x	x	x			x		x	x	x				x	
2B Cosmopolitanism				x	x		x	x					x					
2C Peer Pressure																		
2D External policies and incentives				x		x	x	x	x					x	x		x	x
**3 Inner Setting**																		
3A Structural Characteristics		x	x	x	x		x	x				x	x	x	x			
3B Networks and Communications		x		x	x	x		x	x	x	x	x	x		x	x		x
3C Culture		x	x	x		x	x	x			x	x	x			x	x	x
3D Implementation Climate																		
	Tension for change	x	x	x		x			x	x	x			x				
	Task focused approach to care							x	x			x	x		x	x	x	x
	Compatibility	x				x	x		x		x	x					x	
	Relative Priority	x					x								x			
	Organisational Incentives																	
	Goals and Feedback		x			x				x								
	Learning Climate		x	x								x						x
3E Readiness for Implementation	Leadership Engagement		x									x						

For peer review only

Suppl Table 2 Healthcare Professional Participants: Saturation table and interview characteristics

**Participants n=17**		**1**	**2**	**3**	**4**	**5**	**6**	**7**	**8**	**9**	**10**	**11**	**12**	**13**	**14**	**15**	**16**	**17**
	Available Resources	x	x	x	x	x	x	x	x	x	x	x	x	x	x	x	x	x
	Access to knowledge and info		x	x	x	x		x	x	x	x		x				x	x
**4 Characteristics of Individuals**																		
Knowledge and beliefs		x	x	x		x		x	x	x			x		x		x	x
Self-efficacy		x	x	x		x	x		x	x	x		x		x	x		
	Ability of patients to self-complete	x	x	x	x	x	x	x	x	x	x	x	x	x	x	x	x	x
Individual stage of change	Previous experience of PROMs usage	x	x	x	x	x	x	x	x	x	x		x	x	x	x	x	
Individual identification with organisation			x	x						x	x		x					
Other personal attributes		x	x	x	x		x	x				x	x	x	x	x	x	
**5 Process**																		
5A Planning						x		x										
5B Engaging						x	x		x									x
	Opinion Leaders	x				x	x				x							x
	Formally appointed leaders	x		x	x	x		x	x	x	x		x		x			x
	Champions		x	x				x			x		x					x
	External Change Agents												x			x		x
	Key Stakeholders					x												
	Innovation Participants					x												
5C Executing																		
5D Reflecting and Evaluating																x		

First time data coded to this node in NVIVO	x
Nothing coded to this node in NVIVO	

For peer review only

PROM-HD Study

Using patient reported outcome measures (PROMs) to promote quality of care and safety in the management of patients with established kidney disease requiring treatment with haemodialysis

Topic Guide – Patient Participant Interviews

Research Objectives

To explore the feasibility of using routine PROM assessment to aid the management of patients with established renal failure undergoing HD

Respondent Profile

18 years or over, patients who are receiving renal replacement therapy in the form of HD, who meet the study inclusion/exclusion criteria

Logistics

Face-to face interviews: place, date, and time (start and end) of the interviews to be recorded.

Telephone interviews: write the name, title, telephone number of the respondent and record data and time (start and end time) of interview.

Introduction

Introduce self as a Nurse Researcher but explain that whilst I have a nursing background I am not part of their direct healthcare team, so please feel to talk openly and honestly. Participants should be reassured about the confidential nature of the interview and that all comments will be anonymised before use.

Topics and areas of questioning

A: Awareness of PROMs

1. Have you ever completed questionnaires about your QoL or experiences of receiving dialysis before?

2. Having looked at the sample questionnaires – do they capture the things you think are most important about living with kidney disease and being on dialysis

3. Do you think the collection of PROM questionnaires would be beneficial? Do you think the capture of these outcomes is important?

B. Practical Aspects around PROM collection

1. Would you be able to complete a PROM questionnaire successfully on your own, and if not, how much assistance do you think you would require?

2. How often should they be completed and how long should completion take?

For peer review only - http://bmjopen.bmj.com/site/about/guidelines.xhtml

For peer review only

3. Where and when would you want to complete the PROMs i.e. at home? At the Unit? Before or during dialysis?

4. How should PROM data be collected i.e. in paper or electronic formats such as mobile app or telephone recognition systems?

C. Feedback of PROM data

1. How would you like to receive feedback from the clinical team on your PROM data? i.e. email, letter, graphs in person etc.

D. Barriers or facilitators

1. What do you think are the likely factors (barriers and facilitators) that may improve or discourage the completion of PROMs?

2. What do you think are the issues about PROM collection in different patient groups (e.g. older patients, patients of non-white ethnicity)?

E. Use of PROMs in Research

1. Have you have been involved in PROM collection for research purposes?

2. Should PROMs that collect data for routine clinical and research purposes be different?

3. Do you think any of the practical considerations change when you are collecting PROMs for research rather than as part of routine practice? i.e. would you be prepared to complete them more often for research

4. Are there any special considerations to be given to feedback when using PROMs in a research setting?

For peer review only

For peer review only - http://bmjopen.bmj.com/site/about/guidelines.xhtml

PROM-HD Study

Using patient reported outcome measures (PROMs) to promote quality of care and safety in the management of patients with established kidney disease requiring treatment with haemodialysis

Topic Guide – HCP Interviews

Research Objectives

To explore the feasibility of using routine PROM assessment to aid the management of patients with established renal failure undergoing HD

Respondent Profile

Healthcare Professionals (HCPs) who meet the study inclusion/exclusion criteria.

Logistics

Interview: place, date, and time (start and end) of the interviews to be recorded.

Introduction

Introduce self as a Nurse Researcher but explain that whilst I have a nursing background I am not part of the direct healthcare team, so please feel to talk openly and honestly. Participants should be reassured about the confidential nature of the interview and that all comments will be anonymised before use.

Topics and areas of questioning

A: Awareness of PROMs

1. Have you ever been involved in the collection of PROMs or data regarding QoL and patient experience before?

   Do you think the collection of PROM questionnaires would be beneficial?

   Do you think the capture of these outcomes is important? What do you think about the current measures available and the use of core outcome sets.

   Do you think they could help focus a consultation? Should a 'good' clinician already be able to elicit those outcomes that are important to a patient? Would PROMs alter the way a clinician talks or engages with a patient?

   If yes, how, do you think they could help improve care for a group of patients?

   Do you think they could assist in the multidisciplinary team (MDT) care/follow up of the patient?

   How much time do you spend talking to patients in a typical consultation? Would you like to spend more/less?

2. Having looked at the sample questionnaires – do they capture the things you think are most important about living with kidney disease and being on dialysis from your

For peer review only - http://bmjopen.bmj.com/site/about/guidelines.xhtml

perspective as a MDT member?

a. Are there specific aspects of dialysis care that you think would be effected by the use of PROMs

b. How do you think PROMs differ from PREMs? Do you think collection of one is more important than the other?

B. Practical Aspects around PROM collection

1. Do you think most patients would be able to complete a PROM questionnaire successfully on their own, and if not, how much assistance do you think they would require and from whom? Could assistance affect the responses?
2. How often should they be completed and how long should review of the results take?
3. Where and when would you want the PROMs completed i.e. at home? Before or during dialysis?
4. How should PROM data be collected i.e. in paper or electronic formats such as mobile app or telephone recognition systems?

C. Feedback of PROM data

1. Who would be the best member of the MDT to review the data initially? What about alerts? How should they be managed? Some staff said they don't have time, is that true?
2. What might be the best way the feedback the PROM data to the (a) clinical team, (b) patient? i.e. email, letter, graphs in person etc.
3. How to do you deliver your QA results?
4. How do you feel dealing with problems that you can't solve – do you feel there are areas outside your area of expertise you would not want to discuss? Is listening enough?
5. Specifically, what PROM data do you think would be important within (a) a clinic environment, (b) an MDT meeting, and how might it be presented? E.g. line graphs, bar charts, raw tabulated data etc.
6. Could they be used to show longitudinal trends? Do you think they would be able to pick up ceiling effects or sensitive changes?

D. Barriers or facilitators

1. What do you think are the likely factors (barriers and facilitators) that may improve or discourage the completion of PROMs? IT issues/time? Importance of leadership during any implementation phase
2. Is there a risk we are going to overburden patients with multiple questionnaires? Have you heard of CAT/item banking?

For peer review only - http://bmjopen.bmj.com/site/about/guidelines.xhtml

3. What is the best way to implement into your workflow – could they affect your work flow patterns?

4. Would you want educating about PROMs – what form would that learning take? What specifically would you want more information on? Theory behind collection of PROS, Core outcome sets? Measures available, how they may be used in routine care?
   Are there opportunities to undertake advanced communications training?

5. What do you think about are the key issues about PROM collection in different patient groups (e.g. older patients, patients of non-white ethnicity)?

E. Use of PROMs with patients undergoing Home HD

1. In your opinion, how do patients dialysing at home differ from those dialysing in satellite units? Are they better at 'coping', dealing with change or is it their support structures? Do you think there is utility measuring PROs with this group of patients in particular? Role of carers in this group, how much should they be involved in the process?

2. Would any of your answers differ for patients undergoing Home HD in terms of when, where, how often, who should review and provide feedback?

3. Do you think Home HD patients are more likely to engage with digital technology?

F. Use of PROMs in Research

1. Have you have been involved in PROM use for research purposes? do you know how the measurement tool was chosen? Do you think it captured outcomes that were relevant for the research project?

2. Do you think it would be a good idea to use core outcome sets for renal trials? Should outcomes and the associated PROM that collects them differ between use for routine clinical and research purposes?

3. Do you think any of the practical considerations change when you are collecting PROMs for research rather than as part of routine practice? i.e. how often and where they are completed

4. Are there any special considerations to be given to feedback when using PROMs in a research setting?

For peer review only - http://bmjopen.bmj.com/site/about/guidelines.xhtml

COREQ (COnsolidated criteria for REporting Qualitative research) Checklist

A checklist of items that should be included in reports of qualitative research. You must report the page number in your manuscript where you consider each of the items listed in this checklist. If you have not included this information, either revise your manuscript accordingly before submitting or note N/A.

Topic	Item No.	Guide Questions/Description	Reported on Page No.
**Domain 1: Research team and reflexivity**			
Personal characteristics			
Interviewer/facilitator	1	Which author/s conducted the interview or focus group?	
Credentials	2	What were the researcher's credentials? E.g. PhD, MD	
Occupation	3	What was their occupation at the time of the study?	
Gender	4	Was the researcher male or female?	
Experience and training	5	What experience or training did the researcher have?	
Relationship with participants			
Relationship established	6	Was a relationship established prior to study commencement?	
Participant knowledge of the interviewer	7	What did the participants know about the researcher? e.g. personal goals, reasons for doing the research	
Interviewer characteristics	8	What characteristics were reported about the inter viewer/facilitator? e.g. Bias, assumptions, reasons and interests in the research topic	
**Domain 2: Study design**			
Theoretical framework			
Methodological orientation and Theory	9	What methodological orientation was stated to underpin the study? e.g. grounded theory, discourse analysis, ethnography, phenomenology, content analysis	
Participant selection			
Sampling	10	How were participants selected? e.g. purposive, convenience, consecutive, snowball	
Method of approach	11	How were participants approached? e.g. face-to-face, telephone, mail, email	
Sample size	12	How many participants were in the study?	
Non-participation	13	How many people refused to participate or dropped out? Reasons?	
Setting			
Setting of data collection	14	Where was the data collected? e.g. home, clinic, workplace	
Presence of non-participants	15	Was anyone else present besides the participants and researchers?	
Description of sample	16	What are the important characteristics of the sample? e.g. demographic data, date	
Data collection			
Interview guide	17	Were questions, prompts, guides provided by the authors? Was it pilot tested?	
Repeat interviews	18	Were repeat inter views carried out? If yes, how many?	
Audio/visual recording	19	Did the research use audio or visual recording to collect the data?	
Field notes	20	Were field notes made during and/or after the inter view or focus group?	
Duration	21	What was the duration of the inter views or focus group?	
Data saturation	22	Was data saturation discussed?	
Transcripts returned	23	Were transcripts returned to participants for comment and/or	

For peer review only

Topic	Item No.	Guide Questions/Description	Reported on Page No.
		correction?	
**Domain 3: analysis and findings**			
Data analysis			
Number of data coders	24	How many data coders coded the data?	
Description of the coding tree	25	Did authors provide a description of the coding tree?	
Derivation of themes	26	Were themes identified in advance or derived from the data?	
Software	27	What software, if applicable, was used to manage the data?	
Participant checking	28	Did participants provide feedback on the findings?	
Reporting			
Quotations presented	29	Were participant quotations presented to illustrate the themes/findings? Was each quotation identified? e.g. participant number	
Data and findings consistent	30	Was there consistency between the data presented and the findings?	
Clarity of major themes	31	Were major themes clearly presented in the findings?	
Clarity of minor themes	32	Is there a description of diverse cases or discussion of minor themes?	

Developed from: Tong A, Sainsbury P, Craig J. Consolidated criteria for reporting qualitative research (COREQ): a 32-item checklist for interviews and focus groups. *International Journal for Quality in Health Care*. 2007. Volume 19, Number 6: pp. 349 – 357

Once you have completed this checklist, please save a copy and upload it as part of your submission. DO NOT include this checklist as part of the main manuscript document. It must be uploaded as a separate file.

For peer review only

BMJ Open

Using Patient Reported Outcome Measures during the management of patients with end stage kidney disease requiring treatment with haemodialysis (PROM-HD): a qualitative study.

Journal:	*BMJ Open*
Manuscript ID	bmjopen-2021-052629.R1
Article Type:	Original research
Date Submitted by the Author:	21-Jun-2021
Complete List of Authors:	Anderson, Nicola; University of Birmingham; University Hospitals Birmingham NHS Foundation Trust<br>McMullan, Christel; University of Birmingham, Institute of Applied Health Research<br>Calvert , Melanie ; University of Birmingham, Centre for Patient Reported Outcomes Research and Institute of Applied Health Research<br>Dutton, Mary; University Hospitals Birmingham NHS Foundation Trust, Renal Medicine<br>Cockwell, Paul; University Hospitals Birmingham NHS Foundation Trust, Department of Renal Medicine<br>Aiyegbusi, Olalekan ; University of Birmingham, Centre for Patient-Reported Outcomes Research; University of Birmingham, NIHR Birmingham Biomedical Research Centre, University of Birmingham and University Hospitals Birmingham NHS Foundation Trust<br>Kyte, Derek; University of Worcester
**Primary Subject Heading**:	Renal medicine
Secondary Subject Heading:	Qualitative research, Communication, Patient-centred medicine
Keywords:	NEPHROLOGY, End stage renal failure < NEPHROLOGY, QUALITATIVE RESEARCH, Telemedicine < BIOTECHNOLOGY & BIOINFORMATICS, Dialysis < NEPHROLOGY

SCHOLARONE™
Manuscripts

I, the Submitting Author has the right to grant and does grant on behalf of all authors of the Work (as defined in the below author licence), an exclusive licence and/or a non-exclusive licence for contributions from authors who are: i) UK Crown employees; ii) where BMJ has agreed a CC-BY licence shall apply, and/or iii) in accordance with the terms applicable for US Federal Government officers or employees acting as part of their official duties; on a worldwide, perpetual, irrevocable, royalty-free basis to BMJ Publishing Group Ltd ("BMJ") its licensees and where the relevant Journal is co-owned by BMJ to the co-owners of the Journal, to publish the Work in this journal and any other BMJ products and to exploit all rights, as set out in our licence.

The Submitting Author accepts and understands that any supply made under these terms is made by BMJ to the Submitting Author unless you are acting as an employee on behalf of your employer or a postgraduate student of an affiliated institution which is paying any applicable article publishing charge ("APC") for Open Access articles. Where the Submitting Author wishes to make the Work available on an Open Access basis (and intends to pay the relevant APC), the terms of reuse of such Open Access shall be governed by a Creative Commons licence – details of these licences and which Creative Commons licence will apply to this Work are set out in our licence referred to above.

Other than as permitted in any relevant BMJ Author's Self Archiving Policies, I confirm this Work has not been accepted for publication elsewhere, is not being considered for publication elsewhere and does not duplicate material already published. I confirm all authors consent to publication of this Work and authorise the granting of this licence.

For peer review only

Qualitative Study – Manuscript

Using Patient Reported Outcome Measures during the management of patients with end stage kidney disease requiring treatment with haemodialysis (PROM-HD): a qualitative study.

Nicola Anderson MSc, RN Dip HE[1,2], Christel McMullan Phd[1,3], Melanie Calvert PhD[1,3,4,6,7], Mary Dutton MSc[2], Paul Cockwell PhD[5], Olalekan L Aiyegbusi PhD [1,4,6,7] Derek Kyte PhD[1,8]

[1] Institute of Applied Health Research, Centre for Patient Reported Outcomes Research, University of Birmingham, Birmingham, United Kingdom

[2]Research and Development, University Hospitals Birmingham NHS Foundation Trust

[3] NIHR SRMRC, University Hospitals Birmingham NHS Foundation Trust and University of Birmingham, UK

[4] NIHR Birmingham Biomedical Research Centre, University Hospitals Birmingham NHS Foundation Trust and University of Birmingham, UK

[5] Department of Nephrology, University Hospitals Birmingham NHS Foundation Trust

[6] Birmingham Health Partners Centre for Regulatory Science and Innovation

[7] NIHR Applied Research Collaboration West Midlands, University of Birmingham

[8] School of Allied Health and Community, University of Worcester, Worcester, UK

Corresponding author: Nicola Anderson NEA451@bham.ac.uk

For peer review only - http://bmjopen.bmj.com/site/about/guidelines.xhtml

ABSTRACT

Objectives

Patients undergoing haemodialysis report elevated symptoms, reduced health-related quality of life, and often prioritise improvements in psychosocial wellbeing over long-term survival. Systematic collection and use of patient reported outcomes (PROs) may help support tailored healthcare and improve outcomes. This study investigates the methodological basis for routine PRO assessment, particularly using electronic formats (ePROs), to maximise the potential of PRO use, through exploration of the experiences, views and perceptions of patients and Healthcare Professionals (HCPs) on implementation and use of PROs in haemodialysis settings.

Study design

Qualitative study

Setting and participants

Semi structured interviews with 22 patients undergoing haemodialysis, and 17 HCPs in the United Kingdom.

Analytical Approach

Transcripts were analysed deductively using the Consolidated Framework for Implementation Research (CFIR) and inductively using Thematic Analysis.

Results

For effective implementation, the potential value of PROs needs to be demonstrated empirically to stakeholders. Any intervention must remain flexible enough for individual and

For peer review only - http://bmjopen.bmj.com/site/about/guidelines.xhtml

aggregate use, measuring outcomes that matter to patients and clinicians, while maintaining operational simplicity.

Any implementation must sit within a wider framework of education and support for both patients and clinicians who demonstrate varying previous experience of using PROs and often confuse related concepts. Implementation plans must recognise the multidimensionality of end stage kidney disease and treatment by haemodialysis, while acknowledging the associated challenges of delivering care in a highly specialised environment.

To support implementation, careful consideration needs to be given to barriers and facilitators including effective leadership, the role of champions, effective launch, and ongoing evaluation.

Conclusions

Using the CFIR to explore the experiences, views and perceptions of key stakeholders, this study identified key factors at organisational and individual levels which could assist effective implementation of ePROs in haemodialysis settings. Further research will be required to evaluate subsequent ePRO interventions to demonstrate impact and benefit to the dialysis community.

Key Words:

Haemodialysis, End Stage Kidney Disease, Patients Reported Outcomes, Electronic Patient Reported Outcomes, Health Related Quality of Life

For peer review only - http://bmjopen.bmj.com/site/about/guidelines.xhtml

Article Summary

Strengths and limitations of this study

- Qualitative methods yield rich data with face to face semi-structured interviews allowing the interviewer to monitor non-verbal communications and clarify ambiguous responses.

- This study involved explorations of a prospective intervention, meaning some participants were unfamiliar with key concepts. Pre-interview materials were shared to support and inform discussion and participation.

- The role of the researchers was carefully considered, to acknowledge and minimise bias associated with beliefs and values. Steps were taken to mitigate risks, including use of reflective diaries, participant checking and multiple researchers involved in the coding process.

- While purposive sampling methods led to a diverse sample of participants, it is acknowledged that the sample did not include non-English speakers or carers. Further research is required.

- Data was collected before the coronavirus pandemic. The healthcare delivery landscape in the UK has changed, and it is possible that some attitudes and beliefs particularly around digital data capture may have evolved.

For peer review only - http://bmjopen.bmj.com/site/about/guidelines.xhtml

Funding Statement

This presentation refers to independent research funded by the National Institute of Health Research (NIHR) under its Clinical Doctoral Research Fellowship Programme (Grant Reference ICA-CDRF-2018-04-ST2-027)

Competing interests

MC is a NIHR Senior Investigator and receives funding from the NIHR Birmingham Biomedical Research Centre, the NIHR Surgical Reconstruction and Microbiology Research Centre and NIHR Applied Research Collaboration West Midlands at the University of Birmingham and University Hospitals Birmingham NHS Foundation Trust, Health Data Research UK, Innovate UK (part of UK Research and Innovation), Macmillan Cancer Support, UCB Pharma and GSK. MC has received personal fees from Astellas, Takeda, Merck, Daiichi Sankyo, Glaukos, GSK and the Patient-Centered Outcomes Research Institute (PCORI) outside the submitted work. DK reports grants from Macmillan Cancer Support, Innovate UK, the NIHR, NIHR Birmingham Biomedical Research Centre, and NIHR SRMRC at the University of Birmingham and University Hospitals Birmingham NHS Foundation Trust, and personal fees from Merck and GSK outside the submitted work. O.L.A. is funded by the NIHR BRC, Birmingham and declares personal fees from Gilead Sciences Ltd. and GSK. The remaining authors declare no competing interests.

The views expressed are those of the author(s) and not necessarily those of the NHS, the NIHR or the Department of Health. The study sponsor and funders have no role in study design, including collection, management, analysis and interpretation of data; writing of the report and the decision to submit the report for publication.

For peer review only - http://bmjopen.bmj.com/site/about/guidelines.xhtml

INTRODUCTION

The prevalence of end stage kidney disease (ESKD) requiring treatment with renal replacement therapies such as haemodialysis (HD) continues to rise worldwide (1). Both underlying disease and treatment are associated with a high symptom burden and reduced health-related quality of life (HRQoL) (2, 3). Historically, outcomes such as mortality and dialysis adequacy based on biomedical parameters have been used to inform the management of dialysis services and individual care (4). However, there is now a body of established research (5-8) (9, 10) demonstrating the importance of also capturing patient-reported outcomes (PROs). PRO measures capture data directly from patients on how they feel or function, without requiring interpretation from others , using standardised symptom and/or QoL questionnaires (11), and are often in electronic format (ePROs).

However, while Patient-Reported Experience Measures (PREMs), which allow patients to self-report their experience of receiving healthcare, have been collected since 2017 (12), PROs are still not routinely and systematically collected to manage individual patient care in the United Kingdom: meaning that many patients and some members of the multidisciplinary team caring for them are inexperienced in PROs and related concepts.

Yet the international body of evidence exploring the use of PROs in nephrology settings is growing (13-22), demonstrating acceptability and feasibility of ePROs capture (23-25) and how ePROs can support the delivery of patient centred-care (26, 27). However, the overall impact and benefit of PROs use in dialysis care is yet to be established (28). In comparison, PROs research in oncology has shown defined benefits ranging from improved QoL and reduction in hospitalisations to overall survival (29) and even cost-effectiveness (30).

For peer review only - http://bmjopen.bmj.com/site/about/guidelines.xhtml

For peer review only

In Nov 2020, an online UK Summit led by the UK Renal Association, entitled 'ePROs for the Kidney Patient Community' was held to create a comprehensive, UK-wide roadmap to facilitate and optimize the collection and use of ePROs for the benefit of people with chronic kidney disease. This summit highlighted the importance of key stakeholder engagement, including patients and frontline clinicians, early and at all stages of design and implementation (31).

To inform and direct future research plans, this study aimed to investigate the methodological basis for routine PROs assessment through exploration of the experiences, views and perceptions of patients and HCPs on implementation and use of PROs, particularly ePROs, in haemodialysis settings.

METHODS

DESIGN

The qualitative research question: 'What are the experiences, views and perceptions of patients undergoing HD on the implementation and use of ePROs in routine care and research settings?'

Epistemologically, pragmatism, which is set within a paradigm of enquiry processes and research practicality (32), provides the philosophical framework to answer this question. A core assumption of pragmatism is that research should proceed from a wish to produce actionable knowledge (33); allowing the researcher to select the research design and the methodology deemed most appropriate (34). These foundations led to the decision to use the Consolidated Framework for Interventional Research (35) to offer a theoretical perspective and a Qualitative Descriptive (QD) methodology (36-38). This is particularly useful for healthcare studies focused on discovering the who, what, and where of events or experiences and gaining understanding of inadequately understood phenomenon. Kim et al (38) describe

For peer review only - http://bmjopen.bmj.com/site/about/guidelines.xhtml

QD as the ‘label of choice’ when an unambiguous description of a phenomenon is desired or information is sought to develop and refine questionnaires or interventions (39). This choice of methodology led to the utilisation of the following methods:

Participant Selection:

Ethics approval was issued on 13 September 2017 (Ref No: 17/WA/0281).

Participants included adults (≥18years), receiving HD (in-centre or at home) able to provide valid informed consent and converse in everyday English. Patients were excluded if they were not deemed established on HD or had been undergoing HD < 3 months, or if they had an active intercurrent medical problem requiring enhanced routine clinical care (40). Participants were identified and recruited between July 2018 and November 2019 by the lead author. Eligible patients were primarily approached face to face in the dialysis unit; Patients dialysing in-centre were approached pre dialysis session and patients who dialysed at home (HHD) were approached when they attended for clinic. Purposive sampling was undertaken to achieve maximum variation across age, gender, ethnicity, time on dialysis and co-morbidities (41). HCPs were recruited from the broader renal team, and initially contacted by email by the lead author, they included Health Care and Administrative Assistants (40).

Setting:

Data was collected from 22 patients undergoing haemodialysis and 17 HCPs in the United Kingdom (see tables 1 and 2). All participants were being treated via, or working at, a large regional hospital. To accurately reflect the diversity within the dialysis population, patients being treated in-centre (n=15) were recruited from two satellite units as well as 7 patients choosing to dialyse at home. Non-medical members of the multidisciplinary team were targeted from these two units, one in a city and one serving mainly rural communities. All

For peer review only - http://bmjopen.bmj.com/site/about/guidelines.xhtml

HCPs currently working in the home setting (n=5) had extensive previous experience of in-centre dialysis delivery.

Data Collection:

All consenting participants took part in an audio-recorded semi-structured interview with the lead author, either face-to-face or by telephone. Ethical approval was gained for follow up interviews, but none were required. Patient interviews were conducted in the dialysis unit for those dialysing in-centre (n=15) and at home for the HHD group (n=7). Since these interviews were conducted in patient homes, family members were sometimes present at the request of the patient, but did not take an active role in the interview. HCPs were interviewed in a private room at their workplace. Topic guides were used to steer and support the interview process (see Supplementary boxes 1 and 2). These guides were piloted with one participant from each group and then refined iteratively during the collection phase in response to initial findings. Field notes and in-depth memos were created after each interaction. Since PROs are not routinely collected in the UK, including the hospital trust where this research was undertaken, it was recognised pre-interview information was required to aid the quality of discussion around PROs and future implementation. Therefore participants were provided with a diagram of a core outcome set selected specifically for use in HD trials, to illustrate the position of PROs in outcome measurement (42) and example PROs from a recent systematic review and supported by our local Renal Public and Patient Involvement group: (Kidney Disease Quality of Life SF (KDQOL-SF) (43), Kidney Disease Quality of Life 36 (KDQOL-36) (44) and Integrated Patient Outcome Scale – Renal (IPOS-Renal)) (45). Patients in all UK Renal Units are invited to complete the annual Renal PREM to report their experience of kidney care. This report demonstrates variation in experience across centres and can be used to drive organisational change and improvement (12) ; some patient participants indicated they had completed the annual PREM.

For peer review only - http://bmjopen.bmj.com/site/about/guidelines.xhtml

Analysis

Using Codebook Thematic Analysis (46), a coding framework, drawing upon the Consolidated Framework for Implementation Research (CFIR) (35), was used to deductively analyse transcripts, with further new subthemes developed inductively through data engagement and the analytic process.

The CFIR is a widely utilised conceptual framework developed to guide systematic assessment of factors that might influence implementation and effectiveness, including assessing potential barriers and facilitators in preparation for implementing an innovation (See Figure 1) (35, 47). Primary data analysis was conducted by the lead author, with two investigators (CMcM, DK) reviewing coding for consistency and appropriateness. As the lead author was a renal research nurse conducting this research as part of a Clinical Doctoral Research Fellowship, she was known to some HCP participants. This information was declared and discussed during the valid informed consent process. Additionally a reflective research diary, memo-writing and discussion with the study management team were used to try to minimise the influence of prior relationships upon analysis. Data analysis was supported by Qualitative Data Analysis Software – QSR NVivo v12. Participant verification (48), to check that the transcript correctly documented the discussion, was undertaken on one transcript from each group – no discrepant comments were reported. Data collection and analysis continued until saturation was achieved, i.e. no new information pertinent to the research question was being generated by further interviews (see Suppl Saturation Tables 1 and 2) (49).

Patient and Public Involvement (PPI)

Research on the use and implementation of PROs in nephrology settings was prioritised by the local Kidney Patients Charity. An existing PPI group was utilised and a study specific PPI

For peer review only - http://bmjopen.bmj.com/site/about/guidelines.xhtml

group was convened to help develop the research question, design this study and the associated Fellowship application. Patients were consulted on the study documentation, including topic guides and example materials (see Supplementary box 1 and 2). A summary of study findings will be made available for study participants.

RESULTS

Participant characteristics

Participant characteristics are summarised in Tables 1 and 2.

The patient sample was broadly representative of prevalent HD population in the UK (50) .

Data Saturation was deemed achieved after interviewing 16 HCPs and 15 patients (see supplementary tables 1 and 2 for further detail).

Key Findings

Analysis identified the following themes, which are presented in line with the CFIR key domains. Although presented in a linear fashion, the five domains and their respective constructs cannot be considered in isolation, all interact to effect implementation.

Figure 1 shows the conceptual framework and associated findings. Illustrative quotations are provided in Box 1.

1. Intervention Characteristics: Factors associated with design and quality of the intervention, including how intervention is perceived.

The importance of demonstrating PRO efficacy and impact

Both patients and HCP participants felt that PROs could support the delivery of person-centred care through shared decision making and management in dialysis settings. However,

For peer review only - http://bmjopen.bmj.com/site/about/guidelines.xhtml

HCP interviewees highlighted the key challenge was not the physical collection of PROs, but how the data was then used to improve outcomes. For patients, it was key that any time spent completing questionnaires should be rewarded with review and appropriate action, i.e., not a 'tick-box' exercise.

HCPs, particularly nephrologists, extolled the importance of PROs in research settings.

Intervention flexibility

The topic guide initiated discussions with participants on key practical considerations, including frequency (of PRO completion), optimal timing (around dialysis), preferred setting (home or in-centre), favoured mode of administration (electronic or paper versions) and interpretation and feedback of the responses.

It was clear from patients that a fixed means of implementing PROs would not meet the requirements of this heterogeneous group. Any system would need to be flexible whilst maintaining maximum simplicity. Most patients felt they could complete electronic measures if required, but some strongly favoured paper options. Most interviewees stated that self-completion was possible but felt some would need physical or emotional support from carers. A few patients indicated a desire for assistance with IT aspects until they became familiar. Several patients alluded to the need to 'compartmentalise' their dialysis, by conducting all dialysis-related activity in-centre or by having discrete facilities and times for dialysis if being treated at home; suggesting that PRO completion would need to be undertaken at defined times/settings within their schedule. Others were less concerned about daily reminders of their disease and management, so were open to completion timings/settings that were more variable.

HCPs discussed the promise of electronic capture to support enhanced management of chronic symptoms, but some expressed concerns about potentially missing acute signs and

For peer review only - http://bmjopen.bmj.com/site/about/guidelines.xhtml

effectiveness of associated safety reporting and actions. Two HHD patients foresaw the potential benefits of automated safety alerts associated with electronic capture. However, one patient noted that it was important that any automated information or self-help advice should not conflict with information given face to face by the doctor.

Participants were asked for their perspectives on Computer Adaptive Technology (CAT), a type of assessment in which questions are generated specifically for each individual, using Item Response Theory. This was a new concept to nearly all participants, but most quickly grasped the underlying theory after a short explanation and was supportive of the idea, recognising the potential to stop redundant questions and save time. (See Box 2 for overview of practical considerations).

2. Outer Setting: the wider societal, economic, and organisational context in which the stakeholders and organisation implementing the intervention reside.

Identifying outcomes that matter

There was general agreement amongst all participants that it was important to ensure measurement of 'what outcomes matter most' to patients (51). There was broad agreement from patients that the example PROs they had been provided with were comprehensive in covering the key symptoms facing patients undertaking HD. Furthermore, they agreed with findings from the SONG-HD initiative (42) that fluctuations in HRQoL mattered more, to them, than biomedical outcomes. The importance of these outcomes were acknowledged by the HCPs, but it was felt that deep exploration of some PRO data fell outside the remit of the nephrologist or dialysis nurse and there was anxiety about acting outside their competency and the associated risks of litigation.

The 'Bigger Picture'

For peer review only - http://bmjopen.bmj.com/site/about/guidelines.xhtml

Several patients highlighted problems with continuity of care and lack of cohesion between primary care and their dialysis provision. One HHD patient felt the use of PROs could assist uptake of home therapies, by demonstrating better overall outcomes/HRQoL for patients managing their own dialysis. Participants in both groups reflected that PROs could help support shared decision making by targeting and prioritising discussions according to the patient's agenda.

3. Inner Setting: the structural and cultural contexts around where the implementation will take place

Compatibility of PROs use and approaches to care in HD settings.

Discussing potential implementation and how participants perceive the current situation as needing change, patients and staff alike discussed the approaches to care within dialysis settings. Patients clearly described a 'task orientated' style of care in-centre, with a focus on practically administering HD, versus a more patient-centred model discussed by those dialysing at home. HHD Patients dialysing themselves felt they had developed a level of expertise and the increased 'control' had improved their HRQoL. They indicated current communication pathways were effective and that the HHD team was responsive and holistic in their approach to care. Analysis of patient and HCP data suggested that time constraints were the major reason for a task-based approach in-centre with both patients and staff exhibiting a desire to complete dialysis sessions with as little impediment as possible. Correspondingly most in-centre patients did not feel staff had time to have long discussions and some suggested PROs might help rectify this. There was no discernible variation in the views of the HHD cohort on the practicalities of PRO collection.

Part of a wider framework: The 'information loop', the role of PROs in addressing training needs and health literacy

For peer review only - http://bmjopen.bmj.com/site/about/guidelines.xhtml

HCP data analysis highlighted the importance of education, training, and support for successful implementation. It was indicated that while medical staff needed training in PRO interpretation, such as rating scales, nurses might need a broader program on use of PROs as well as associated chronic disease management. There was disparity indicated in the health literacy of the patient group; some HHD patients appeared expert in their illness and treatment, whilst some in-centre patients appeared less confident in their knowledge.

4. Characteristics of Individuals: Factors associated with individuals involved in implementation

Previous experience of using PROs

Patients expressed an overall lack of awareness and experience of PROs. Many had completed annual PREMs but highlighted a lack of feedback or action. Some patients expressed general anxiety about questionnaire completion, linked to their experience of externally administered questionnaires for other agencies: for example, Personal Independence Payment (PIP) surveys assessing entitlement to extra costs associated with long term ill health or disability. When reviewing the example PROs, it was noted that some patients, particularly those from minority ethnic backgrounds, flagged the potential difficulty around asking about sensitive issues such as sexual function. Whilst the participants themselves were happy to discuss such issues, they recognised that not all patients might be.

All nephrologists interviewed had experience of using PROs in research settings, but none had regularly and systematically collected PROs in routine care. PROs use in any setting was largely a new concept for nursing staff.

There was confusion around the concepts of PROs versus PREMs, expressed by both patients and HCPs. Participants found it hard to distinguish experience of care from outcomes and used terms interchangeably.

For peer review only - http://bmjopen.bmj.com/site/about/guidelines.xhtml

Multidimensional aspects of ESKD and consequent challenges

Many of the patients in the study had multiple co-morbidities and associated symptoms, reflective of the wider HD population. Patients indicated that it was often hard to know which symptoms related to their ESKD and/or treatment, and which were associated with other diseases or advancing age; leading to anxiety about what to raise during a consultation. It was suggested that PROs data review and feedback gave a chance to discuss symptoms and potential causes, thereby potentially providing reassurance. However, some clinicians thought it was outside their role to manage non-nephrology related outcomes presented by PROs and that it would be difficult to disentangle several, possibly unrelated, symptoms. They feared focusing on symptoms that were not currently being experienced or intractable could cause frustration or anxiety for themselves and patients. Some patients gave a contrasting opinion, revealing the chance to be heard, even without a solution, was often sufficient to maintain the patient-clinician relationship. There was also concern that an over reliance on PROs could result in a distraction from other important clinical issues.

The experience of life on dialysis was highly varied, with some participants exhibiting signs of depression, anger, or acceptance, as well as reporting multiple symptoms.

5. The Process of Implementation

Supporting the process of implementation

The analysis identified leadership, the identification of champions and a 'bottom up' approach to communication and shared solutions among both patients and clinicians as key factors supporting implementation. All participants emphasised the importance of support during completion, however, patients highlighted this need not always be delivered by health care staff. Peer-to-peer support and non-clinical champions i.e., administrative staff, might assist. HCPs felt a comprehensive launch was important and that individual roles should be

For peer review only - http://bmjopen.bmj.com/site/about/guidelines.xhtml

clear. They reflected that senior members of the clinical team would be opinion leaders, and any change agents would need to understand the dialysis setting. Evaluation and reflection were important process components, to recognise and deal with any unintended consequences.

Overarching themes: Barriers and Facilitators to effective implementation

Across all CFIR constructs, analysis identified potential barriers and facilitators to the introduction of PROs.

Potential barriers

Nephrologists cited a lack of evidence base supporting the use of PROs in routine kidney care and were concerned regarding the risk of over-medicalising the patient experience. There were perceived time barriers for staff i.e., workflow interruptions, additional obligations caused by PROs; with nurses perceiving quality time with patients in-centre as limited. HCP participants argued that nursing documentation had lessened available time, as it often required recording on digital devices away from the bedside. They feared patients were being overburdened by questionnaires, particularly the less health literate.

In contrast, patients were often already aware of the complications associated with ESKD and HD. Many had achieved a degree of acceptance and some were reassured that the symptoms were expected and not something new to deal with. Frustration would only arise if, having taken time to complete questionnaires, no action was taken. Both groups agreed that information technology issues i.e., inability to log in, no Wi-Fi could be a barrier.

Potential facilitators

Several HCPs considered how PROs might work to deliver a relative advantage over current systems to support more patient-centred care. Participants felt PROs could be a

For peer review only - http://bmjopen.bmj.com/site/about/guidelines.xhtml

communication tool – acting like a ‘tin opener’; but requiring that responses be carefully probed, highlighting that this required appropriate skills and training. Others saw the measures as an Aide Memoire which could help target the consultation and even save time; as could the possibility of remote management of care using ePROs. Perhaps most importantly, PROs were seen by both patient and HCPs as a way to get to know the patient and hence deliver more meaningful care.

DISCUSSION

There is a growing body of published, peer-reviewed literature exploring the experiences of both kidney patients and multidisciplinary kidney HCP with ePROs (13, 17, 23, 26, 27, 52). Previous studies have included non-dialysis dependent Chronic Kidney Disease (CKD) and peritoneal dialysis populations (17, 23, 27), evaluating existing ePROs and associated delivery systems. This paper adds to the corpus by using semi-structured interviews to gain rich data to inform and optimise future ePRO implementation in a HD population naïve to ePRO collection and clinicians unused to routine and systematic ePRO use.

The key findings of this study are, that while patients and HCPs support the concept of PROs, further evidence of their potential benefit are required for effective implementation. It emphasises that any system should be flexible and measure what matters most to patients. Most importantly the data collected should be acted upon. There was a general lack of awareness and experience of PROs particularly among patients and nurses, with concern amongst the HCPs that PRO capture may highlight issues they might have neither the experience nor resources to manage. Therefore, a comprehensive implementation strategy is required to support any delivery, which involves strong leadership, patient and clinician involvement and ongoing training.

For peer review only - http://bmjopen.bmj.com/site/about/guidelines.xhtml

This study highlighted the importance of getting ‘buy in’ i.e., gaining acceptability, from these stakeholders. This could be achieved by demonstrating evidence on their potential benefits thereby increasing trust to warrant practice change. Clinicians particularly questioned whether positive effects on survival, reported QoL, patient-clinician communications and cost efficiencies demonstrated in oncology (29, 53-55) could be replicated in HD settings.

The body of evidence around ePROs in nephrology is emerging. Studies from North America demonstrate the feasibility of electronic capture of PROs in HD (19, 24) and an Australian pilot study is currently exploring the feasibility and acceptability of ePROs capture and feedback among patients receiving HD in the Symptom monitoring With Feedback Trial (20). In advanced CKD populations, the RePROM study is piloting the use of an ePRO for remote symptom monitoring in the UK (56), whilst the AmbuFlex telepro system is used to manage renal follow up in Denmark (25). Aiyegbusi et al. (2019) explored key stakeholder perspectives on the use of PROs in these pre-dialysis patients and their findings are consistent with this study, particularly regarding potential benefits and administrative aspects (17); suggesting PRO data collection early in the patient pathway can be instituted and continue as illness advances through to renal replacement therapy or even conservative care.

A review and synthesis of evidence is currently being undertaken to investigate how PROs might work to enhance patient-centred care in renal settings, to offer strategy and guidance at individual and aggregate levels of decision-making (57). For effective multiple uses of data, implementation needs to be viewed within the context of complex data linkage and accessibility issues. Studies in the UK and Australia are currently exploring and testing such linkage of symptoms and QoL data to the Australia and New Zealand Dialysis and Transplant Registry and UK Renal Registry (20, 22, 58).

For peer review only - http://bmjopen.bmj.com/site/about/guidelines.xhtml

However, the purpose of data collection must be clear. Participants in this study confused the concepts of PROs versus PREMs, understandable given that experience of undertaking HD is intricately linked to both HRQoL and symptoms. The integrated use of both measures, side by side, warrants further investigation. Anxieties about purpose and data sharing could affect patient engagement; clear communication of roles and expectations should be undertaken.

Whilst guidance exists on what outcomes to measure in research (5, 6) and routine practice (9) this has not necessarily been centred on routine capture and feedback to guide individual care. Consequently, it is not clear which measure(s) should be used to capture data in a non-burdensome way, whilst providing sufficient measurement properties to support both single patient monitoring and aggregation of data where required, meaning further enquiry is needed.

The general lack of awareness and experience of PROs and concern that PROs capture may highlight issues that HCPs should not or cannot not deal with, leads to a significant finding that PROs implementation must sit within a broader educational framework. PROs could be used to support wider initiatives and training, especially in the nursing group, who can then cascade information and self-management skills to patients, thereby increasing overall patient health literacy. This could be considered as closing an 'information loop'. There is already evidence that patient education is associated with better patient outcomes (59) and new strategies and quality improvement programs exist such as Shared HD, a UK programme aiming to support patients receiving in-centre haemodialysis to be more independent and confident in participating in aspects of their own care (60). Potential barriers are cost and resources, but increasingly, accredited online learning facilities are available. However, it is acknowledged, staff in this study indicated a preference for face-to-face training. Education and Training on PROs would be needed for all stakeholders.

For peer review only - http://bmjopen.bmj.com/site/about/guidelines.xhtml

This study identified time and workflow interruptions as key barriers to implementation. Rotenstein et al. (2017) found that following initiation of routine PROs collection in surgical settings, such concerns shifted as clinicians became comfortable with new processes. There was even a suggestion that PROs could enhance physician satisfaction and prevent 'burnout' (61). Tong et al. (2018) interviewed nephrologists, who also identified resource constraints and uncertainties in how to prioritize, measure, and manage a range of competing comorbidities and broader quality of life outcomes in a clinical setting that is technically demanding and traditionally focused on biochemical factors (62); findings mirrored by this study. Such anxieties will need addressing.

During the SARS-CoV 2 pandemic, data from the UK Renal Registry showed that patients undergoing HD experience a relative risk of mortality of 45.5% compared to the general population (63). This vulnerable population has been unable to effectively shield, requiring regular dialysis treatment with associated risks around shared transport and waiting areas. Various strategies have been employed to reduce risks (64) but new ways of care delivery were required. The pandemic has irrevocably changed healthcare, with increased use of virtual services (65) and remote clinic visits. The addition of ePROs to readily available biomedical data means that clinicians could more effectively deliver patient-centred care without the necessity of the patient being physically present. This study also suggests that remote symptom monitoring could offer patients the safety and confidence to dialyse at home and arguably ePROs could assist the HomeHD service in maintaining its patient-centred approach while serving ever increasing numbers.

There are limitations to this study. Participants needed to be English speakers; this could affect transferability of findings. Views of carers were not specifically sought but their influence and importance were clearly identified by patients, suggesting further research targeting this group is warranted. Critically, data collection took place pre-SARS-COV-2

For peer review only - http://bmjopen.bmj.com/site/about/guidelines.xhtml

pandemic and experiences and perceptions around digital data capture and new approaches to healthcare delivery may well have shifted.

To conclude, the SARs-COV-19 pandemic has caused an irreversible shift in healthcare delivery, with increased use of digital communication and assessment. While the nephrology community, both patients and HCPs, are largely supportive of the concept of ePROs, there remain caveats to their routine and systematic use. Stakeholders need to be convinced by empirical evidence, considering the best available measures and methodological considerations. By exploring the experiences, views and perceptions of major stakeholders, this study identified key factors at organisational and individual levels which would assist effective implementation of ePROs. Further research will then be required to evaluate any subsequent ePROs interventions to empirically demonstrate impact and benefit of their use to the dialysis community.

Author's Contributions:

NA, CMcM, MC, MD, PC, OLA, DK: Substantial contribution to the conception and design of the study
NA: Data collection
NA, CMcM, DK Analysis and interpretation of data for the work
NA, CMcM, MC, MD, PC, OLA, DK: Drafting and revising the work critically for important intellectual content **and** final approval of version to be published **and** agreement to be accountable for all aspects of the work in ensuring that questions related to the accuracy or integrity of any part of the work are appropriately investigated and resolved.

Acknowledgments:

PROM-HD PPI Group

Renal Research Patient Advisory Group

Queen Elizabeth Hospital Kidney Patients Association (QEKPA)

For peer review only - http://bmjopen.bmj.com/site/about/guidelines.xhtml

Data Availability Statement:
De identified data may be available upon reasonable request via the corresponding author (ORCID 0000-0002-0614-3198).

Protocol available doi:10.1136/bmjopen-2018-021532.

Ethical Approval Statement:
Ethics approval was issued on 13 September 2017 (Ref No: 17/WA/0281). Wales 7 Ethics Committee.

For peer review only

REFERENCES:

1. Bikbov B, Purcell CA, Levey AS, Smith M, Abdoli A, Abebe M, et al. Global, regional, and national burden of chronic kidney disease, 1990–2017: a systematic analysis for the Global Burden of Disease Study 2017. The Lancet. 2020;395(10225):709-33.
2. Almutary H, Bonner, A, Douglas, C. Symptom burden in Chronic Kidney Disease: a review of recent literature. Journal of Renal Care. 2013;39(3):140-50.
3. Abdel-Kader K, Unruh ML, Weisbord SD. Symptom burden, depression, and quality of life in chronic and end-stage kidney disease. Clinical Journal of The American Society of Nephrology: CJASN. 2009;4(6):1057-64.
4. KDIGO. KDIGO 2012 Clinical Practice Guideline for the Evaluation and Management of Chronic Kidney Disease. Kidney International Supplements 2013;3(1).
5. Evangelidis N, Tong A, Manns B, Hemmelgarn B, Wheeler DC, Tugwell P, et al. Developing a Set of Core Outcomes for Trials in Hemodialysis: An International Delphi Survey. Am J Kidney Dis. 2017. 70 (4) pages 564-575
6. Tong A, Manns B, Hemmelgarn B, Wheeler DC, Evangelidis N, Tugwell P, et al. Establishing Core Outcome Domains in Hemodialysis: Report of the Standardized Outcomes in Nephrology-Hemodialysis (SONG-HD) Consensus Workshop. Am J Kidney Dis. 2017;69(1):97-107.
7. Viecelli AK, Tong A, O'Lone E, Ju A, Hanson CS, Sautenet B, et al. Report of the Standardized Outcomes in Nephrology-Hemodialysis (SONG-HD) Consensus Workshop on Establishing a Core Outcome Measure for Hemodialysis Vascular Access. Am J Kidney Dis. 2018. 71 (5) Pages 690-700
8. Urquhart-Secord R, Craig JC, Hemmelgarn B, Tam-Tham H, Manns B, Howell M, et al. Patient and Caregiver Priorities for Outcomes in Hemodialysis: An International Nominal Group Technique Study. Am J Kidney Dis. 2016;68(3):444-54.
9. Verberne WR, Das-Gupta Z, Allegretti AS, Bart HAJ, van Biesen W, Garcia-Garcia G, et al. Development of an International Standard Set of Value-Based Outcome Measures for Patients With Chronic Kidney Disease: A Report of the International Consortium for Health Outcomes Measurement (ICHOM) CKD Working Group. Am J Kidney Dis. 2019;73(3):372-84.
10. International Consortium for Health Outcomes Measurement (ICHOM). Chronic Kidney Disease: Data Collection Reference Guide. ICHOM; 2017 Oct 25th 2017. Contract No.: Version 1.0. https://www.ichom.org/wp-content/uploads/2020/02/ICHOM-Chronic-Kidney-Disease-Reference-Guide-20171025-1.00.pdf Last Accessed 20.01.21
11. FDA. Guidance for industry. Patient reported outcomes measures: Use in medical product development to support labeling claims. 2009. Docket No:FDA-2006-D-0362. https://www.fda.gov/media/77832/download Last Accessed 20.01.21
12. UK Renal Assocation & Kidney Care UK. Patient Reported Experience of Kidney Care in the UK 2020. Published 2021. https://www.kidneycareuk.org/news-and-campaigns/prem/#:~:text=PREM%20stands%20for%20Patient%20Reported%20Experience%20Measures.%20The,improvement%20can%20be%20made%20from%20a%20patient%20perspective Last accessed 14 June 2021.
13. Smith V, Wise K. Evaluating nurses' action outcomes and exploring their perspectives of implementing the POS-S (Renal) assessment tool for haemodialysis patients. Renal Society of Australasia Journal. 2017;13(1):14-21.
14. Schick-Makaroff K, Mharapara P. Use of Electronic Patient-Reported Outcomes in the Care of Patients with Kidney Failure. Nephrology Nursing Journal: Journal of the American Nephrology Nurses' Association. 2020;47(5):465-72.
15. Aiyegbusi OL, Kyte D, Cockwell P, Marshall T, Dutton M, Slade A, et al. Using Patient-Reported Outcome Measures (PROMs) to promote quality of care and safety in the management of patients with Advanced Chronic Kidney disease (PRO-trACK project): a mixed-methods project protocol. BMJ Open. 2017;7(6):e016687.

For peer review only - http://bmjopen.bmj.com/site/about/guidelines.xhtml

16. Aiyegbusi OL, Kyte D, Cockwell P, Marshall T, Dutton M, Walmsley-Allen N, et al. Development and usability testing of an electronic patient-reported outcome measure (ePROM) system for patients with advanced chronic kidney disease. Computers in Biology & Medicine.2018 101:120-7.
17. Aiyegbusi OL, Kyte D, Cockwell P, Marshall T, Dutton M, Walmsley-Allen N, et al. Patient and Clinician Perspectives on Electronic Patient-Reported Outcome Measures in the Management of Advanced CKD: A Qualitative Study. Am J Kidney Dis. 2019. 74 (2) pages 167-178
18. Kyte D, Anderson N, Auti R, Aiyegbusi OL, Bishop J, Bissell A, et al. Development of an electronic patient-reported outcome measure (ePROM) system to aid the management of patients with advanced chronic kidney disease. J Patient Rep Outcomes. 2020;4(1):55.
19. Gabbard J, McLouth CJ, Brenes G, Claudel S, Ongchuan S, Burkart J, et al. Rapid Electronic Capturing of Patient-Reported Outcome Measures in Older Adults With End-Stage Renal Disease: A Feasibility Study. Am J Hosp Palliat Care. 2020:1049909120954805.
20. Viecelli A, Dansie K, McDonald S, Jesudason S, Duncanson E, Bennett P, et al. Symptom monitoring with feedback trial (SWIFT) pilot to explore the feasibility and acceptability of electronic patient reported outcome measures (e-proms) data capture and feedback. Nephrology Dialysis Transplantation. 2020;35 (SUPPL 3):iii137.
21. Breckenridge K, Bekker HL, Gibbons E, van der Veer SN, Abbott D, Briancon S, et al. How to routinely collect data on patient-reported outcome and experience measures in renal registries in Europe: an expert consensus meeting. Nephrol Dial Transplant. 2015;30(10):1605-14.
22. Van Der Veer SN, Ercia A, Caskey FJ, Farrington K, Jury F, Rees M, et al. Developing an Intervention to Implement Electronic Patient-Reported Outcomes in Renal Services in the UK. Studies in Health Technology & Informatics. 2020;270:936-40.
23. Schick-Makaroff K, Molzahn AE. Evaluation of real-time use of electronic patient-reported outcome data by nurses with patients in home dialysis clinics. BMC Health Serv Res. 2017;17(1):439.
24. Schick-Makaroff K, Molzahn A. Strategies to use tablet computers for collection of electronic patient-reported outcomes. Health & Quality of Life Outcomes. 2015;13:2.
25. Schougaard L M LLP, Jessen A et al. AmbuFlex: tele-patient-reported outcomes (telePRO) as the basis for follow up in chronic and malignant diseases. Qual Life Res. 2016;25(3):525-34.
26. Trillingsgaard C NBK, Hjollund N H, Lomborg K. Use of patient-reported outcomes in outpatient settings as a means of patient involvement and self-management support - a qualitative study of the patient perspective. European Journal for Patient-Centred Healthcare. 2016;4(2):359-67.
27. Schick-Makaroff K, Tate K, Molzahn A. Use of Electronic Patient Reported Outcomes in Clinical Nephrology Practice: A Qualitative Pilot Study. Canadian Journal of Kidney Health & Disease. 2019;6:2054358119879451.
28. Yang Y, Chen H, Qazi H, Morita PP. Intervention and Evaluation of Mobile Health Technologies in Management of Patients Undergoing Chronic Dialysis: Scoping Review. JMIR Mhealth Uhealth. 2020;8(4):e15549.
29. Basch E, Deal AM, Dueck AC, Scher HI, Kris MG, Hudis C, et al. Overall Survival Results of a Trial Assessing Patient-Reported Outcomes for Symptom Monitoring During Routine Cancer Treatment. JAMA. 2017;318(2):197-8.
30. Lizée TB, Basch E ; Trémolières, P ; Voog, E ; Domont, J ; Peyraga, G ; Urban, T ; Bennouna, J ; Septans, A ; Balavoine, M ; Detournay, B ; Denis, F. Cost-Effectiveness of Web-Based Patient-Reported Outcome Surveillance in Patients With Lung Cancer. Journal of Thoracic Oncology. 2019;14(6):1012-21.
31. UK Renal Association . Electronic Patient-Reported Outcomes (ePROs) for the kidney patient community 2020. Available at: https://wwwyoutubecom/watch?v=JgG61Vouctk&feature=youtube and https://wwwyoutubecom/watch?v=iQ0mOVee_dg&feature=youtube[Accessed Jan 2021]
32. Kelly LM, Cordeiro M. Three principles of pragmatism for research on organizational processes. Methodological Innovations. 2020;13(2).

For peer review only - http://bmjopen.bmj.com/site/about/guidelines.xhtml

33. Feilzer Y M. Doing Mixed Methods Research Pragmatically: Implications for the Rediscovery of Pragmatism as a Research Paradigm. Journal of Mixed Methods Research. 2009;4(1):6-16.
34. Kaushik V, Walsh CA. Pragmatism as a Research Paradigm and Its Implications for Social Work Research. Social Sciences. 2019;8(9).
35. Damschroder LJ, Aron DC, Keith RE, Kirsh SR, Alexander JA, Lowery JC. Fostering implementation of health services research findings into practice: a consolidated framework for advancing implementation science. Implement Sci. 2009;4:50.
36. Sandelowski M. Whatever happened to qualitative description? Research in Nursing & Health. 2000;23:334-40.
37. Sandelowski M. What's in a name? Qualitative description revisited. Res Nurs Health. 2010;33(1):77-84.
38. Kim H, Sefcik JS, Bradway C. Characteristics of Qualitative Descriptive Studies: A Systematic Review. Res Nurs Health. 2017;40(1):23-42.
39. Neergaard M, Olesen F, Andersen RS, Søndergaard J. Qualitative description - the poor cousin of health research? BMC Medical Research Methodology. 2009;9:52
40. Anderson NE, Calvert M, Cockwell P, Dutton M, Aiyegbusi OL, Kyte D. Using patient-reported outcome measures (PROMs) to promote quality of care in the management of patients with established kidney disease requiring treatment with haemodialysis in the UK (PROM-HD): a qualitative study protocol. BMJ Open. 2018;8(10):e021532.
41. Etikan I. Comparison of Convenience Sampling and Purposive Sampling. American Journal of Theoretical and Applied Statistics. 2016;5(1).
42. Standardised Outcomes in Nephrology (SONG) . The SONG Handbook. Version 1. Sydney, Australia 2017. Available from: songinitiative.org/reports-and-publications/.Last accessed 20.01.2021
43. Hays R D KJD, Mapes D L, Coons S J, Amin N, Carter W B, Kamberg C. Kidney Disease Quality of Life Short Form (KDQOL-SF): A Manual for use and scoring. In: RAND, editor. 1997. https://www.rand.org/content/dam/rand/pubs/papers/2006/P7994.pdf Last Accessed 20.01.2021

44. Hays RD, Kallich JD, Mapes DL, Coons SJ, Carter WB. Development of the kidney disease quality of life (KDQOL) instrument. Quality of Life Research. 1994;3(5):329-38.
45. Pos-pal.org.. POS Palliative Care Outcome Scale 2017 [Available from: https://pos-pal.org/maix/ipos-renal-in-english.php. Last Accessed 20.01.2021
46. Braun V Clarke V. Can I use TA? Should I use TA? Should I not use TA? Comparing reflexive thematic analysis and other pattern-based qualitativeanalytic approaches. Counselling & Psychotherapy Research. 2020https://doi.org/10.1002/capr.12360

47. van Oers HA, Teela L, Schepers SA, Grootenhuis MA, Haverman L, PROMs I, et al. A retrospective assessment of the KLIK PROM portal implementation using the Consolidated Framework for Implementation Research (CFIR). Qual Life Res. 2020. https://doi.org/10.1007/s11136-020-02586-3
48. Birt L, Scott S, Cavers D, Campbell C, Walter F. Member Checking: A Tool to Enhance Trustworthiness or Merely a Nod to Validation? Qual Health Res. 2016. June 22 https://doi.org/10.1177/1049732316654870

49. Kerr CN, A. Wild, D. Assessing and demonstrating data saturation in qualitative inquiry supporting patient-reported outcomes research. Expert Review of Pharmacoeconomics & Outcomes Research. 2010;10(3):269-81.
50. UK Renal Registry . UK Renal Registry 22nd Annual Report – data to 31/12/2018,. Bristol, United Kingdom; 2020. https://renal.org/audit-research/22nd-annual-report-data-31122018 Last accessed 14 June 2021.
51. Coulter A. Measuring what matters to patients. BMJ. 2017;356:j816.

For peer review only - http://bmjopen.bmj.com/site/about/guidelines.xhtml

52. Tong A, Winkelmayer WC, Wheeler DC, van Biesen W, Tugwell P, Manns B, et al. Nephrologists' Perspectives on Defining and Applying Patient-Centered Outcomes in Hemodialysis. Clin J Am Soc Nephrol. 2017;12(3):454-66.
53. Denis F, Basch EM, Lethrosne C, Pourel N, Molinier O, Pointreau Y, et al. Randomized trial comparing a web-mediated follow-up via patient-reported outcomes (PRO) vs. routine surveillance in lung cancer patients: Final results. Journal of Clinical Oncology. 2018;36(15_suppl):6500
54. Denis F, Basch, E, Septans, A, Bennouna, J, Urban,T, Dueck, A, Letellier, C. Two year survivial comparing web-based symptom monitiring vs routine surveillance following treatment for lung cancer. JAMA. 2019;321(3):306-7.
55. Greenhalgh J AP, McCluskey S, Takeuchi E, Velikova G. How do doctors refer to Patient Reported Outcome Measures (PROMs) in oncology consultations. Quality of Life Research. 2013;22(5):939-50.
56. Kyte D, Bishop J, Brettell E, Calvert M, Cockwell P, Dutton M, et al. Use of an electronic patient-reported outcome measure in the management of patients with advanced chronic kidney disease: the RePROM pilot trial protocol. BMJ Open. 2018;8(10):e026080.
57. Schick-Makaroff K, Thummapol O, Thompson S, Flynn R, Karimi-Dehkordi M, Klarenbach S, et al. Strategies for incorporating patient-reported outcomes in the care of people with chronic kidney disease (PRO kidney): a protocol for a realist synthesis. Syst Rev. 2019;8(1):20.
58. Morton RL, Lioufas N, Dansie K, Palmer S, Jose M, Raj R, et al. Patient reported outcome measures in australian and new zealand renal units: An anzdata survey of current practice. Nephrology.23:32.2019: https://doi.org/10.1111/nep13577
59. Narva AS, Norton JM, Boulware LE. Educating Patients about CKD: The Path to Self-Management and Patient-Centered Care. Clin J Am Soc Nephrol. 2016;11(4):694-703.
60. Wilkie M, Barnes T. Shared Hemodialysis Care: Increasing Patient Involvement in Center-Based Dialysis. Clin J Am Soc Nephrol. 2019;14(9):1402-4.
61. Rotenstein LS, Huckman RS, Wagle NW. Making Patients and Doctors Happier — The Potential of Patient-Reported Outcomes. New England Journal of Medicine. 2017;377(14):1309-12.
62. Tong A, Winkelmayer WC, Wheeler DC, Biesen WV, Tugwell P, Manns B, et al. Nephrologists' perspectives on defining and applying patient-centered outcomes in hemodialysis. Clinical Journal of the American Society of Nephrology. 2017;12(3):454-66.
63. Savino M, Casula A, Santhakumaran S, Pitcher D, Wong E, Magadi W, et al. Sociodemographic features and mortality of individuals on haemodialysis treatment who test positive for SARS-CoV-2: A UK Renal Registry data analysis. PLoS One. 2020;15(10):e0241263.
64. Roper T, Kumar N, Lewis-Morris T, Moxham V, Kassimatis T, Game D, et al. Delivering Dialysis During the COVID-19 Outbreak: Strategies and Outcomes. Kidney Int Rep. 2020;5(7):1090-4.
65. Hull SA, Rajabzadeh V, Thomas N, Hoong S, Dreyer G, Rainey H, et al. Do virtual renal clinics improve access to kidney care? A preliminary impact evaluation of a virtual clinic in East London. BMC Nephrol. 2020;21(1):10.
66. Charlson M E, Ales K L, Mackenzie C R. A new method of classifying prognostic comorbidity in longitudinal studies: Development and validation J Chron Dis 1987;40(5):373-83.

For peer review only - http://bmjopen.bmj.com/site/about/guidelines.xhtml

Box One: Key Findings – Summary of direct quotes

Key Finding	Illustrative Quotation	
**1. Intervention Characteristics**	**Patient Participants**	**HCP Participants**
**The importance of demonstrating efficacy/benefit**	*'If they haven't got a point for collecting them that isn't going to be actioned or put into a specific piece of research, then they shouldn't be there……'Yes, it makes you unwilling to invest your own time in something that you hadn't seen any benefit from in the past….If it's just a box ticking exercise for your unit or whatever, to say that I've complied with some directive and nothing is going to change, then why would you.' Patient JI18*	*'one is convincing those patients and doctors and nurses that it's [PROs] actually of use to man or beast. I think that is going to be the main barrier'. HCP11.*<br><br>*'I think if you could then see that it changed a consultation that meant that you went, 'Oh, I didn't know you were having that issue; I can do something about that'. The difficulty is if they then, it drives a lot of conversations that, you know, saying, 'Yeah, can't really do anything about then', and then everybody may well think well this hasn't changed anything so, you know, is it making you feel better writing it down? Don't know really. So if there's not an outcome difference to it then it just becomes a kind of form-filling exercise but there's not obviously a benefit'. HCP16*
**The intervention must be flexible/offer choices**	*'Yeah, but certainly filling it in is not an issue, I think discussing it and whether somebody has their partner with them or carer or whatever, I think it's something you should offer them and it's for them to make up their mind' Patient MN07*	*'I guess we have to give the option to the patient whether and how they want to fill this in or not because some patients may not want to fill it in. And I can think of a few patients on my dialysis unit that probably would not want to fill this in.'HCP14*
**Practical considerations**	See Box 3 for key findings and illustrative direct quotes.	

For peer review only - http://bmjopen.bmj.com/site/about/guidelines.xhtml

**2. Outer Setting**		
**Identifying and measuring Outcomes that matter**	*I need to go out. I need to keep this going. I need to keep feeling that I'm worth something by bringing home a wage and working' Patient YZ13*<br><br>*'It does really knock the quality of life, some people tend to be more worried about the quantity of life but I'm interested in the quality of life because not having quality of life, I can understand why some people do commit suicide, there's no quality' Patient ST10*	*Yes, definitely. We see that all the time don't we? I mean you know, we see that all the time. I mean that's the battle, to get people to dialyse three times a week rather than twice a week [yeah, yeah]. So you know, I'm having this battle with a man in XUnit at the moment who's decided to go and dialyse twice a week and he tried it for a few weeks. And he said, 'Oh, I feel perfectly well. So I'm just going to carry on dialysing twice a week' and I said, 'Well, that's not the point' [laughs] [absolutely]. And he said, 'Yeah, but I feel fine. I get more time, I get more spare time'. HCP13*
**How PRO's fit into the 'Bigger Picture'**	Continuity of care and communication with Primary Care:<br>*' Yeah, the other thing I find with my GP for example, now she rang me, I have monthly blood test here and every 12 months you go to the GP and I go to the diabetic clinic, my diabetes is good…….so the doctor (GP) rang up and said, I think you're anaemic, I said well I've had these blood tests at the dialysis centre and nobody phoned up that I was anaemic……' Patient ST10*	*'I think so, I think it [useful for GP's to be able to see the patients results, answers to some of these questions] would because most of this is in the community so it would be very, very useful for the GP's to be able to access all the information' HCP04*
**3. Inner Setting**		
**Compatibility of PROs and approaches to delivery of care in HD settings**	Task centred care versus patient centred care:<br>*'yes, you might be surrounded by them [unit staff] but they might not be asking questions, you might not feel you can ask questions. There might be aspects that you might not necessarily think it is worthwhile asking' Patient JI18*	Task centred care versus patient centred care:<br>*'we don't always see the bigger picture, we're so focused on getting that needle in to get dialysis, that we forget, oh my god, there's a person on the end of that arm…..' HCP12*<br><br>*'in-centre, it's looking at the patient, home haemo it's looking at the person' HCP08*

For peer review only - http://bmjopen.bmj.com/site/about/guidelines.xhtml

For peer review only

		'I think the nurses have got time to get to know the patients to a certain extent as far as medically, their medication is involved. You know, how poorly they are, obviously what they need. Unfortunately, I don't think they have time to carry that a bit further…..' HCP02
**PRO's as part of a wider educational framework – 'the information loop'**	Health Literacy of patients:<br>*Talking about monthly QA reports – ' ….and we all get these, telling us about our potassium, and somebody says to me, do you understand them? I say, I haven't got a clue, he says nor me, I says, all I know, when I go in there, they tell me that's okay, that's okay, that's okay, I said but I haven't got a clue' Patient BA14*	Implementation must be part of a wider framework of training, education, and support:<br>*' this will be exciting if implemented correctly, but there's a whole massive amount of work, it's not just this, it's everything else around it yeah, I think so' HCP17*<br><br>Staff Education requirements:<br>*'So I guess education would be vital, but they're about communication skills, they're about learning what matters in chronic disease management. And it would also be about, because that's burdensome, it's hugely draining to do it day in day out with a similar group of people. And you would need to put in support, raising the education for them is one, raising awareness. But I also think if you are hearing people's symptoms, which are as I say some of them you can't resolve and you're hearing them day in day out, you need somewhere to offload to as well. So it's like, but building a good structure around what's needed. But that doesn't mean don't talk about them.' HCP05*
**4. Characteristics of Individuals**		
Previous experience of using PROs	Have you ever completed anything like these questionnaires before?<br>*'No I don't think so' Patient UV11, Patient BA14*<br>*'No I haven't' Patient WX12*	*'I mean I've used them in various research projects………And that's one of the reasons why I haven't routinely used them in clinical practice because I think they're fine for research, but I haven't*

For peer review only - http://bmjopen.bmj.com/site/about/guidelines.xhtml

	'I think I have, but I can't remember, it must have been some time ago…..I don't think there were as many questions as that one' [KDQOL-SF] Patient DC15	*found one that's hugely clinically, that I feel I'd really want to use.' HCP06*
Concept of PROs versus PREMs	*Interviewer: 'Have you been asked to complete anything like that [exemplar PROs] before?'*<br>*Patient MN20: 'I've done questionnaires before, I think'*<br>*Interviewer: 'Have you? What kind of questions did they ask? Were they similar ones?'*<br>*Patient MN20: 'Most of them have been in here about the staff and stuff about dialysis and how long I've been on it. It was similar'*	*[PROMs & PREMs] I get confused and I think there's crossover and I find it quite difficult to draw a line between experience and outcome. Because for example, the experience of a patient. So, tiredness for example can be strongly affected by a patient experience. So, you have travelled six hours to get there or waited for your transport, your experience, it affects your tiredness which is – I think – I'm not sure always it's a useful distinction' HCP12*<br><br>*'Interviewer: Yeah. So do you think we should be measuring them (PROMs and PREMs) at the same time?*<br>*HCP16: I think that they should be part of the same thing. I think that they should be seen so that we can pick apart whether people are happy with the service that they're getting but also how that interplays with their health and their feelings and their symptoms so I think the two things overlap.' HCP16*
Multidimensionality of ESKD and consequent challenges	*'…obviously the doctors can't cure that, they can't cure that, they can only cure your medical condition, they can't cure your mental condition so that's for you, but they can make it as well, so they give you the positivity to carry on' Patient BA14*<br><br>*'…..it's most of the time that I get anxious with social situations because of my other medical problems. It's not just about my kidney problems and then dealing with the social aspect. It's my*	*'If we can't do much about it, then it's not our problem, sort of thing. That's one of those barriers; I think there's the worry that it uncovers things that are uncomfortable to talk about….' HCP15*<br><br>*Acknowledgement that there are aspects of care that do not fall within doctors 'remit' 'things that aren't necessarily medical, that may relate to social services or other things that are driving a symptom or feeling*

For peer review only - http://bmjopen.bmj.com/site/about/guidelines.xhtml

For peer review only

	kidney problems, plus my other medical problems and then the social situation. I have to deal with my other medical problems before I can even contemplate going out with family or friends…..or shopping and things like that' Patient WX12<br><br>*' The main problem? [with dialysis] Social life. Absolutely. It's not just my social life; it's the wife's social life as well. She's like a nurse to me really. Without wife, I don't know where I'd be. I've got two lads but you can't rely on them to be here' Patient YZ13*	*that relates more to social care, to a dietitian or other members of the team….' HCP16*
**5. Process of implementation**		
Supporting the process of implementation	Importance of leadership and champions:<br>*Yeah, Yeah, I've helped a lot of people. When I used to go to look at their machines, because they send me first to look at the room and whether it can be done, they didn't even know I was on dialysis. They'd look at me and say "you seem to know a lot about this". I said, "yeah, because I'm a dialysis patient" Patient YZ13*	Importance of leadership and champions:<br>*'Time, resources, you know, if someone's not here, say, if it was me implementing it, if I'm not here, I'm thinking, will it get done? It's as simple as that' HCP17*<br><br>*'You might need to bring somebody else in from outside, yeah but that runs its own complications so what's the answer, a patient representative might be an answer, somebody who knows dialysis.' HCP17*<br><br>Bottom up approach*:*<br>*'Often there are new things that are introduced and when you've just got to do it attitude. Now that just gets peoples backs up to start with………have a proper process in place to prepare people' HCP07*
Barriers to effective implementation:<br>Time/attitude/IT problems	*'They [Doctors] haven't got time anymore. We all know that, but it's nice to not be rushed along and ignored' Patient MN20*<br>*' It does annoy me when we have problems with logging in and passwords' Patient WX12*	*'So it has to be easy access for the patient. It's pointless having something they can't actually get to and that's scary or frustrating to get onto, to log on or something, yeah.' HCP05*

For peer review only - http://bmjopen.bmj.com/site/about/guidelines.xhtml

Facilitators to effective implementation:<br>How PROs might deliver a relative advantage over current systems of care delivery	Acts as a communication tool:<br>*'Yeah, yeah, you know, if you had a form to fill in, you're divorced from it because obviously within our human nature we always, we don't want to look vulnerable, we don't want to look vulnerable, right, so therefore, if the doctors say, you might be feeling sick, and you think, well I only get it now and again, to yourself, this is, so you don't mention it but if you mentioned it, he or she can make a diagnosis on that,….' Patient BA14*<br><br>*'I couldn't seem to settle in, i.e. couldn't communicate with the nurses, me as a person, I don't communicate very well' Patient QR09*<br><br>Help target the consultation: save time<br>*'Probably save your doctor from doing some of the work as well……As I say, if I'm not giving information that I need, we're not getting anywhere, are we? Patient AB01*<br><br>Help deliver person-centred care<br>*'If we've got monthly bloods and we've got the information on that at the same time, they can look at that [ePRO] instead of looking at the computer and seeing what's really going on with that person….'Patient AB01*<br><br>*'Even if that doctor is reading that one-on-one, he can look at this... even if it's on a computer, he can look at it and say, 'He's got a problem with that area. Let's go and deal with that.' Patient AB01*<br><br>*'These questionnaires are good as well because a couple of symptoms cropped up that I do get but that I forgot is related to everything. I thought, 'Oh, that's why I get that then. Oh, that's why I get that.' Patient WX12*	Could reduce workload:<br>*'I find it really interesting how people would say that this is an increase in workload, when actually this is things that you need to be looking at anyway. For me this is nothing out of what I'm already looking at in every patient and of course it will be an increase in workload if you don't look at them.' HCP17*<br><br>*Potentially time saving:*<br>*I think the PROMs, certainly one is useful when they fill them in but quite often they haven't filled them in and it's then sometimes difficult to ascertain have they not filled them in because they have got symptoms but they just aren't necessarily engaging or thinking oh therefore I will fill this in or whether they haven't filled it in 'cause they're fine. But then at the same time it's quite easy to ask a patient and say, 'Have you not filled this in?', because actually they're fine and then they'll say, 'Yes, I'm fine. There's nothing to worry about', sort of thing and then in a way you can think well I can, I don't necessarily need to give you targeted questions 'cause you've already got the opportunity to do that. Sometimes it then speeds things up. HCP06*

For peer review only - http://bmjopen.bmj.com/site/about/guidelines.xhtml

Box 2 - Practical Considerations and illustrative direct quotes

**Practical Considerations**	**Key Findings**	
	**Patient Participants**	**HCP Participants**
**Frequency (to allow for accurate recall)**	Wide variation of responses from 1 week to 12 months. Agreement that it needed to match recall whilst allowing for treatment responses. Several patients would complete as frequently as asked – demonstrating no preference.	Wide variation of responses from, as required to 12 months. Focus on practicality i.e. how often they thought patients would complete and time burden rather than sensitivity to changes and recall. Before routine clinic visit was popular response.
**Timing (in relation to dialysis cycle)**	Some patients wanted to complete during dialysis but noted this could be practically difficult, particularly if their access was in dominant arm. Most did not want to complete immediately after dialysis, the majority requiring some recovery time.	*'Horses for courses' HCP11* meaning that people are different and will need different things. In terms of timing, some will need a short amount of time to complete, others will need longer.<br><br>Options, in-centre before or during session or patients choice at home (need time frame for completion? i.e. within 5 day window)<br>General agreement that straight after dialysis patients are very fatigued, so not optimum time for completion.<br>Effects of cognitive status on concentration levels – ability to partially complete and come back later, recall bias will be relevant. Need to consider are you asking about today, last 2 weeks, last month, when designing tool.
**Setting of completion (Home/in-centre)**	Some favoured completion in centre, others preferred to complete at home, particularly if carer assistance required.	Patients needed options (home/Unit) – some patients need assistance so carers could help (unlikely nurses would have time)<br>It was suggested better returns if encouraged to complete at unit: at home patients are 'distracted by life'.<br>*Will responses be different if completing at home – does that matter? 'either way, you're gonna get the reality of their life, so perhaps it doesn't matter' HCP05*

For peer review only - http://bmjopen.bmj.com/site/about/guidelines.xhtml

		Lack of privacy in-centre noted by number of respondents
**Mode of capture**	Varied responses – digital, paper or no preference.<br>Proponents of digital capture displayed no obvious preference for choice of device. Only one patient had no access to a digital device at home.<br>Preference for paper often reflected lack of confidence in ability to complete digitally, a number said they would need assistance to complete electronically<br>*''I feel like my confidence is down and that needs to build back up again before I use the computer.' Patient CD02*	Advantages of electronic collection raised (real time review, less admin, less likely to lose returns, easier to track responses, to review responses/feedback) but option of paper needs to be available, as some patients would be unable to complete digitally, and who would provide this assistance? Some pondered that current digital resources i.e. PatientView are not widely used. IT systems require resource for infrastructure, upkeep and training.
**Readability**	No jargon or medical terms – got to be easy to read and understand.<br>Guidance around more complex constructs<br>Availability in of measure in other languages<br>*'You may find you need to print them in their own Urdu or whatever, so that they do understand them. And that has to be very careful that the question doesn't get lost in the translation' Patient MN07*	Simple and clear rating scales for staff undertaking interpretation.<br>Need to be culturally sensitive/valid and available in languages other than English.
**Length of questionnaire**	Generally, length was not an issue, provided patients could see a purpose to completion. There was a dislike of repetitive questions. Potential time burden of lengthy questionnaires, seen as an inconvenience for carers<br>*'So the other question I was going to ask was were you put off by the length of some of those questionnaires?'*<br>*Interviewee: 'No I wasn't, no, you know I'd put aside the time to do it so no I wasn't, I could see the point of them.' Patient UV11*	Consensus that the shorter the questionnaire, the better.<br>Some commented that current validated PROs were much too lengthy.
**Form of Feedback**	No overall preference: could be letter, email, or discussion. Most happy to use graphical data. However, important that timely feedback given and PROs completion *'not just a tick box exercise'.*<br>Some patients wanted carers involved in feedback process.<br>*'Because I don't do emails and sometimes my eyesight, I can't able to read. Yeah. I prefer in person'. Patient GHO4*	Consensus that electronic feedback is ideal. Responses outlined in letter to patient or face to face in clinic to allow 'probing' – but this requires resource.<br>Agreement that longitudinal data would be advantageous to see trends – preference for graphical rather than tabular form – but scales need to be well thought through, 0-3 not considered

For peer review only - http://bmjopen.bmj.com/site/about/guidelines.xhtml

	The need for timely feedback was made:<br>*Interviewer: So you'd want to see some sort of feedback within two weeks.*<br>*Interviewee: Two weeks and they sort it out. Not leave it for months and months and years and years, no good at all. Patient KL06*<br><br>*'As well as giving helpful information to help you manage your condition, it would also be useful to have information about what causes certain things in order to put your mind at rest, if you know what I mean?' Patient CD02*	'sensitive' enough to be able to show changes – preference for EQ-5D thermometer scale… *'but a 'Blunt tool'- good clinician should pick up sensitive changes before PROs would' HCP11*<br>No time to read lots of free text
**Setting of feedback**	Currently most consultations take place while the patient is dialysing. Patients gave mixed responses to the wish to be seen in private, most did not want additional or extended visits at the dialysis unit and seemed resigned to being seen in open bays.	If medical staff were providing feedback in person, pragmatically best to do this in clinic, acknowledged this can take place at the bedside during dialysis and this may affect
**Who provides feedback**	No overall preference, but one patient felt more comfortable talking to nursing staff.	Variation on responses – some thought medical staff (but concerns raised around time and workload) other thought nurses (some anxiety regarding time constraints and knowledge/experience of junior nursing staff to know how to manage symptoms or issues raised by PROs)<br>*Need clear process for review and feedback – 'can't have 6 nurses going back to patient to discuss responses HCP16'*.

For peer review only - http://bmjopen.bmj.com/site/about/guidelines.xhtml

Table 1: Patient Participant Characteristics

Variable	Count
**Age**	
Age under 40	2/22 (9%)
40-49	3/22 (13%)
50-59	5/22 (23%)
60-69	3/22 (14%)
70-79	6/22 (27%)
Over 80	3/22 (13%)
**Gender**	
Male	12/22 (55%)
Female	10/22 (45%)
**Ethnicity**	
White -British	16/22 (73%)
Black- British	4/22 (18%)
Asian/Asian British	2/22 (9%)
**In centre Dialysis**	15/22 (68%)
Morning sessions	12/15 (80%)
Home HD	7/22 (32%)
**Current Vascular Access**	
Arterio-Venous Fistula	19/22 (86%)
Arterio-Venous Graft	0/22 (0%)
Central Venous Catheter	3/22 (14%)
**Charlson Comorbidity Score**	
<2	1 (4%)
3-4	5 (23%)
5-6	4 (18%)
7-8	6 (27%)
9-10	5 (23%)
11-12	1(4%)
13+	0 (0%)
**Time since dialysis commencement (years)**	
≤ 5	11/22 (50%)
6-10	5/22 (23%)
11-15	3/22 (14%)
16-20	1/22 (4.5%)
>20	2/22 (9%)
Note n=22	

Charlson Comorbidity Index (CCI) (66) quantifies an individual's burden of disease and corresponding 1-year mortality risk. The index adjusts for 17 comorbidities, each one classified with a validated score of 1–6 points, based on the adjusted relative risk of 1-year mortality. The final total score is used to calculate the probability of survival. The index is being used in this context to demonstrate the overall disease burden of the population under study.

For peer review only

Table 2: Health Care Professional Participant Characteristics

Variable	Count
**Gender**	
Male	5/17 (29%)
Female	12/17 (71%)
**Ethnicity (Office of National Statistics Categories)**	
White -British	9/17 (53%)
Other white background	2/17 (12%)
Black- British	1/17 (6%)
Asian/Asian British	1/17 (6%)
Any other Asian background	2/17 (12%)
Black African	1/17 (6%)
White and Black Caribbean	1/17 (6%)
**Role**	
Consultant Nephrologist	5/17 (29%)
Consultant Surgeon	1/17 (6%)
Registered Nurse	9/17 (53%)
Non-registered Healthcare Assistant	1/17 (6%)
Administrative Assistant	1/17 (6%)
**Time working across HD setting (years)**	
≤ 5	1/17 (6%)
6-10	4/17 (24%)
11-15	3/17 (18%)
16-20	2/17 (12%)
>20	7/17 (41%)
Note n=17	

For peer review only - http://bmjopen.bmj.com/site/about/guidelines.xhtml

For peer review only

For peer review only - http://bmjopen.bmj.com/site/about/guidelines.xhtml

Figure 1 – Conceptual Framework and Key Findings v1 30.11.20

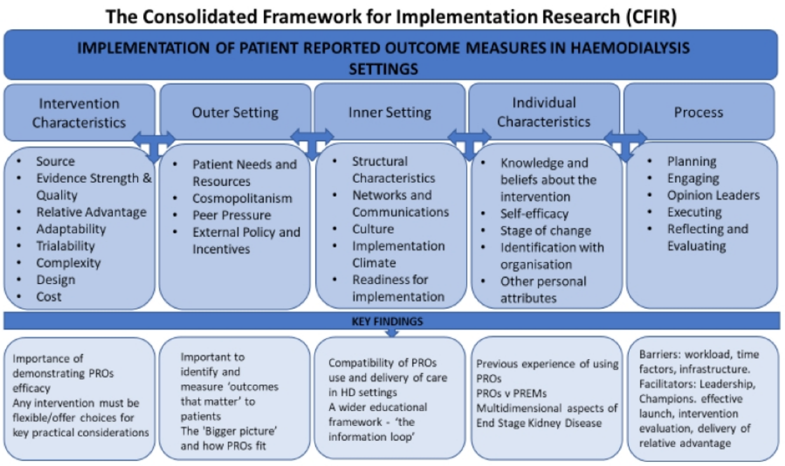

1. Adapted from Damschroder LJ, Aron DC, Keith RE, Kirsh SR, Alexander JA, Lowery JC. Fostering implementation of health services research findings into practice: a consolidated framework for advancing implementation science. Implement Sci. 2009;4:50.

Figure 1 - Conceptual Framework and key findings

297x209mm (300 x 300 DPI)

Supp Table 1 Patient saturation table and interview characteristics

	Value
**Patient Interviews**	
Number invited/Number participating	41/22
Reasons for non-participation	Did not meet eligibility criteria, did not want to participate (transport issues, did not want to do research)
Duration of patient interviews (min)	Range 24-84<br>Mean 50 mins
**Patient Saturation Data**	
No of codes generated	110
Point of data saturation	15th interview
Note n=17	

		1	2	3	4	5	6	7	8	9	10	11	12	13	14	15	16	17	18	19	20	21	22
**1 Innovation Characteristics**																							
1A Innovation Source													x						x				
1B Evidence strength and quality		/	/	/	/	/	/	/	/	/	/	/	/	/	/	/	/	/	/	/	/	/	/
1C Relative Advantage	Perception of advantage	x	x		x	x	x	x			x	x	x		x	x	x		x		x	x	
	Intractable problems								x			x		x	x	x					x		
	Potential to cause anxiety										x			x	x		x			x	x	x	
1D Adaptability	Adaptability of intervention												x						x				
	Use within research	x	x	x	x			x	x			x	x	x		x	x		x				
	Importance of choices in how and what is answered	x	x	x				x					x				x	x				x	
1E Trialability		/	/	/	/	/	/	/	/	/	/	/	/	/	/	/	/	/	/	/	/	/	/
IF Complexity								x											x				
IG Design and Packaging	Frequency	x	x		x		x	x		x	x	x		x	x	x	x			x		x	x
	Length	x	x	x	x		x	x		x	x	x	x	x	x	x	x	x	x	x	x		x
	Language/Readability					x		x	x	x	x	x	x		x		x	x			x		x
	Mode	x	x	x	x	x	x	x	x	x	x	x		x	x	x	x	x	x	x	x	x	x

For peer review only - http://bmjopen.bmj.com/site/about/guidelines.xhtml

Supp Table 1 Patient saturation table and interview characteristics

		1	2	3	4	5	6	7	8	9	10	11	12	13	14	15	16	17	18	19	20	21	22
	Setting	x		x	x	x		x	x		x	x	x	x		x	x	x		x	x	x	x
	Timing	x	x		x	x	x		x	x	x			x	x	x		x	x	x		x	x
	Outcomes that matter	x	x		x	x	x			x			x	x	x	x			x	x		x	
	Current Measures				x	x	x	x	x		x	x	x	x	x		x	x	x		x	x	x
	Safety Alerts	x																	x			x	
	Self-help information		x						x				x	x			x					x	
	ComputerAdaptiveTechnology										x	x		x		x	x		x	x			
	Review and Feedback	x	x		x		x	x	x	x	/	x	x	/	x	x	/	x	x	x		x	x
	Sensitivity to change	/	/	/	/	/	/	/	/	/		/	/		/	/		/	/	/	/	/	/
IH Cost																			x				
**2 Outer Setting**																							
2A Needs and resources	Identification		x	x	x		x	x	x			x	x	x	x			x	x	x	x	x	x
	Barriers	x	x	x	x	x	x	x	x	x	x	x	x	x	x	x	x	x	x	x	x	x	x
	Facilitators	x	x	x	x		x			x	x	x	x	x				x	x	x		x	
2B Cosmopolitanism				x				x			x				x					x			
2C Peer Pressure		/	/	/	/	/	/	/	/	/	/	/	/	/	/	/	/	/	/	/	/	/	/
2D External policies and incentives		/	/	/	/	/	/	/	/	/	/	/	/	/	/	/	/	/	/	/	/	/	/
**3 Inner Setting**																							
3A Structural Characteristics																x							
3B Networks and Communications	Nature and Quality		x	x	x	x	x	x	x	x	x	x	x		x	x		x	x	x	x	x	x
	Shared Decision Making	x	x	x							x	x	x	x	x	x		x		x	x		x
3C Culture											x			x	x	x					x		
3D Implementation Climate	Tension for change		x	x					x			x	x	x	x			x	x	x	x		x
	Compatibility							x				x	x									x	x
	Relative Priority	x			x						x										x	x	
	Organisational Incentives	/	/	/	/	/	/	/	/	/	/	/	/	/	/	/	/	/	/	/	/	/	/

Supp Table 1 Patient saturation table and interview characteristics

		1	2	3	4	5	6	7	8	9	10	11	12	13	14	15	16	17	18	19	20	21	22
	Goals and Feedback	/	/	/	/	/	/	/	/	/	/	/	/	/	/	/	/	/	/	/	/	/	/
	Learning Climate												x										
3E Readiness for Implementation	Leadership Engagement	/	/	/	/	/	/	/	/	/	/	/	/	/	/	/	/	/	/	/	/	/	/
	Available Resources		x		x											x		x				x	
	Access to knowledge and info		x			x	x						x		x	x	x				x	x	x
**4 Characteristics of Individuals**																							
Knowledge and beliefs		x	x	x	x			x	x	x		x		x	x	x	x	x	x	x	x	x	
Self-efficacy	Ability to self-complete	x	x	x		x	x	x	x	x	x	x	x	x	x	x	x	x		x		x	
	Patient as an Expert			x				x				x	x	x	x					x			
	PROMs v PREMs		x		x	x		x											x		x		
Individual stage of change	Previous experience of PROMs usage	x			x	x	x		x	x	x	x	x	x	x	x	x	x	x	x	x	x	x
Individual identification with organisation		x	x		x	x	x	x	x	x	x	x	x	x	x		x			x	x	x	x
Other personal attributes	Multidimensionality of ESKD:																						
	Symptomology	x	x	x	x	x	x	x	x	x	x	x	x	x	x	x	x	x	x	x	x	x	x
	Life on Dialysis	x	x	x	x	x	x	x	x	x	x	x	x	x	x	x	x	x	x	x	x	x	x
**5 Process**																							
5A Planning		/	/	/	/	/	/	/	/	/	/	/	/	/	/	/	/	/	/	/	/	/	/
5B Engaging																							
	Opinion Leaders	/	/	/	/	/	/	/	/	/	/	/	/	/	/	/	/	/	/	/	/	/	/
	Formally appointed leaders					x		x															
	Champions					x							x	x									
	External Change Agents	/	/	/	/	/		/	/	/	/	/	/	/	/	/	/	/	/	/	/	/	/
	Key Stakeholders	/	/	/	/	/		/	/	/	/	/	/	/	/	/	/	/	/	/	/	/	/
	Innovation Participants				x								x										
	Role of Carers	x		x	x		x	x		x			x				x						
5C Executing		/	/	/	/	/	/	/	/	/	/	/	/	/	/	/	/	/	/	/	/	/	/
5D Reflecting and Evaluating		/	/	/	/	/	/	/	/	/	/	/	/	/	/	/	/	/	/	/	/	/	/

Supp Table 1 Patient saturation table and interview characteristics

First time data coded to node in NVIVO	x
Nothing coded to node in NVIVO	/
Nothing coded to node in NVIVO	/

For peer review only

Suppl Table 2 Healthcare Professional Participants: Saturation table and interview characteristics

	Value
**HCP Interviews**	
Number invited/Number participating	19/17
Reasons for non-participation	Competing clinical commitments
Duration of patient interviews (min)	Range 29-63<br>Mean 48 mins
**Patient Saturation Data**	
No of codes generated	134
Point of data saturation	16th Interview
Note n=17	

**Participants n=17**		**1**	**2**	**3**	**4**	**5**	**6**	**7**	**8**	**9**	**10**	**11**	**12**	**13**	**14**	**15**	**16**	**17**
**1 Innovation Characteristics**																		
1A Innovation Source						x										x		
1B Evidence strength and quality		x				x				x		x		x	x	x	x	x
1C Relative Advantage		x	x	x	x	x	x		x	x	x	x	x	x	x	x	x	x
												x	x		x		x	x
	How PROMs might work to deliver advantage		x		x	x	x	x	x	x								
1D Adaptability		x				x												x
	Use within research				x	x							x		x	x	x	
1E Trialability																	x	
IF Complexity		x				x	x		x		x						x	
IG Design and Packaging		x	x	x	x	x	x	x	x	x	x	x	x	x	x	x	x	x
	Frequency	x	x	x	x	x	x	x	x	x	x	x	x	x	x	x		x
	Length			x	x	x	x		x		x	x	x	x	x		x	
	Mode	x	x		x	x			x	x	x	x	x	x		x	x	

For peer review only

Suppl Table 2 Healthcare Professional Participants: Saturation table and interview characteristics

**Participants n=17**		**1**	**2**	**3**	**4**	**5**	**6**	**7**	**8**	**9**	**10**	**11**	**12**	**13**	**14**	**15**	**16**	**17**
	Setting		x	x	x	x		x	x	x	x			x			x	x
	Timing		x	x	x		x		x	x	x	x	x	x	x		x	
	Self-completion		x	x	x	x	x	x				x	x	x	x	x		x
	Outcomes that matter	x	x	x	x		x		x	x	x		x			x		x
	Safety Alerts			x			x				x			x	x	x	x	
	Sensitivity						x		x		x	x						
	Computer Adaptive Technology					x		x	x	x	x	x	x	x		x	x	
	Review and Feedback	x	x	x	x	x	x	x				x	x	x	x	x	x	x
IH Cost														x		x		
**2 Outer Setting**																		
2A Needs and resources						x	x	x		x	x	x	x	x		x		
	Barriers		x		x	x	x	x	x	x	x	x	x	x	x	x	x	x
	Facilitators			x	x	x			x		x	x	x				x	
2B Cosmopolitanism				x	x		x	x					x					
2C Peer Pressure																		
2D External policies and incentives				x		x	x	x	x					x	x		x	x
**3 Inner Setting**																		
3A Structural Characteristics		x	x	x	x		x	x				x	x	x	x			
3B Networks and Communications		x		x	x	x		x	x	x	x	x	x		x	x		x
3C Culture		x	x	x		x	x	x			x	x	x			x	x	x
3D Implementation Climate																		
	Tension for change	x	x	x		x			x	x	x			x				
	Task focused approach to care							x	x			x	x		x	x	x	x
	Compatibility	x				x	x		x		x	x					x	
	Relative Priority	x					x								x			
	Organisational Incentives																	
	Goals and Feedback		x			x				x								
	Learning Climate		x	x								x						x
3E Readiness for Implementation	Leadership Engagement		x									x						

For peer review only

Suppl Table 2 Healthcare Professional Participants: Saturation table and interview characteristics

**Participants n=17**		1	2	3	4	5	6	7	8	9	10	11	12	13	14	15	16	17
	Available Resources	x	x	x	x	x	x	x	x	x	x	x	x	x	x	x	x	x
	Access to knowledge and info		x	x	x	x		x	x	x	x		x				x	x
**4 Characteristics of Individuals**																		
Knowledge and beliefs		x	x	x		x		x	x	x			x		x		x	x
Self-efficacy		x	x	x		x	x		x	x	x		x		x	x		
	Ability of patients to self-complete	x	x	x	x	x	x	x	x	x	x	x	x	x	x	x	x	x
Individual stage of change	Previous experience of PROMs usage	x	x	x	x	x	x	x	x	x	x		x	x	x	x	x	
Individual identification with organisation			x	x						x	x		x					
Other personal attributes		x	x	x	x		x	x				x	x	x	x	x	x	
**5 Process**																		
5A Planning						x		x										
5B Engaging						x	x		x									x
	Opinion Leaders	x				x	x				x							x
	Formally appointed leaders	x		x	x	x		x	x	x	x		x		x			x
	Champions		x	x				x			x		x					x
	External Change Agents												x			x		x
	Key Stakeholders					x												
	Innovation Participants					x												
5C Executing																		
5D Reflecting and Evaluating																x		

First time data coded to this node in NVIVO	x
Nothing coded to this node in NVIVO	

PROM-HD Study

Using patient reported outcome measures (PROMs) to promote quality of care and safety in the management of patients with established kidney disease requiring treatment with haemodialysis

Topic Guide – Patient Participant Interviews

Research Objectives

To explore the feasibility of using routine PROM assessment to aid the management of patients with established renal failure undergoing HD

Respondent Profile

18 years or over, patients who are receiving renal replacement therapy in the form of HD, who meet the study inclusion/exclusion criteria

Logistics

Face-to face interviews: place, date, and time (start and end) of the interviews to be recorded.

Telephone interviews: write the name, title, telephone number of the respondent and record data and time (start and end time) of interview.

Introduction

Introduce self as a Nurse Researcher but explain that whilst I have a nursing background I am not part of their direct healthcare team, so please feel to talk openly and honestly. Participants should be reassured about the confidential nature of the interview and that all comments will be anonymised before use.

Topics and areas of questioning

A: Awareness of PROMs

1. Have you ever completed questionnaires about your QoL or experiences of receiving dialysis before?

2. Having looked at the sample questionnaires – do they capture the things you think are most important about living with kidney disease and being on dialysis

3. Do you think the collection of PROM questionnaires would be beneficial? Do you think the capture of these outcomes is important?

B. Practical Aspects around PROM collection

1. Would you be able to complete a PROM questionnaire successfully on your own, and if not, how much assistance do you think you would require?

2. How often should they be completed and how long should completion take?

For peer review only - http://bmjopen.bmj.com/site/about/guidelines.xhtml

3. Where and when would you want to complete the PROMs i.e. at home? At the Unit? Before or during dialysis?

4. How should PROM data be collected i.e. in paper or electronic formats such as mobile app or telephone recognition systems?

C. Feedback of PROM data

1. How would you like to receive feedback from the clinical team on your PROM data? i.e. email, letter, graphs in person etc.

D. Barriers or facilitators

1. What do you think are the likely factors (barriers and facilitators) that may improve or discourage the completion of PROMs?

2. What do you think are the issues about PROM collection in different patient groups (e.g. older patients, patients of non-white ethnicity)?

E. Use of PROMs in Research

1. Have you have been involved in PROM collection for research purposes?

2. Should PROMs that collect data for routine clinical and research purposes be different?

3. Do you think any of the practical considerations change when you are collecting PROMs for research rather than as part of routine practice? i.e. would you be prepared to complete them more often for research

4. Are there any special considerations to be given to feedback when using PROMs in a research setting?

For peer review only

For peer review only - http://bmjopen.bmj.com/site/about/guidelines.xhtml

PROM-HD Study

Using patient reported outcome measures (PROMs) to promote quality of care and safety in the management of patients with established kidney disease requiring treatment with haemodialysis

Topic Guide – HCP Interviews

Research Objectives

To explore the feasibility of using routine PROM assessment to aid the management of patients with established renal failure undergoing HD

Respondent Profile

Healthcare Professionals (HCPs) who meet the study inclusion/exclusion criteria.

Logistics

Interview: place, date, and time (start and end) of the interviews to be recorded.

Introduction

Introduce self as a Nurse Researcher but explain that whilst I have a nursing background I am not part of the direct healthcare team, so please feel to talk openly and honestly. Participants should be reassured about the confidential nature of the interview and that all comments will be anonymised before use.

Topics and areas of questioning

A: Awareness of PROMs

1. Have you ever been involved in the collection of PROMs or data regarding QoL and patient experience before?

   Do you think the collection of PROM questionnaires would be beneficial?

   Do you think the capture of these outcomes is important? What do you think about the current measures available and the use of core outcome sets.

   Do you think they could help focus a consultation? Should a 'good' clinician already be able to elicit those outcomes that are important to a patient? Would PROMs alter the way a clinician talks or engages with a patient?

   If yes, how, do you think they could help improve care for a group of patients?

   Do you think they could assist in the multidisciplinary team (MDT) care/follow up of the patient?

   How much time do you spend talking to patients in a typical consultation? Would you like to spend more/less?

2. Having looked at the sample questionnaires – do they capture the things you think are most important about living with kidney disease and being on dialysis from your

For peer review only - http://bmjopen.bmj.com/site/about/guidelines.xhtml

perspective as a MDT member?

a. Are there specific aspects of dialysis care that you think would be effected by the use of PROMs

b. How do you think PROMs differ from PREMs? Do you think collection of one is more important than the other?

B. Practical Aspects around PROM collection

1. Do you think most patients would be able to complete a PROM questionnaire successfully on their own, and if not, how much assistance do you think they would require and from whom? Could assistance affect the responses?

2. How often should they be completed and how long should review of the results take?

3. Where and when would you want the PROMs completed i.e. at home? Before or during dialysis?

4. How should PROM data be collected i.e. in paper or electronic formats such as mobile app or telephone recognition systems?

C. Feedback of PROM data

1. Who would be the best member of the MDT to review the data initially? What about alerts? How should they be managed? Some staff said they don’t have time, is that true?

2. What might be the best way the feedback the PROM data to the (a) clinical team, (b) patient? i.e. email, letter, graphs in person etc.

3. How to do you deliver your QA results?

4. How do you feel dealing with problems that you can’t solve – do you feel there are areas outside your area of expertise you would not want to discuss? Is listening enough?

5. Specifically, what PROM data do you think would be important within (a) a clinic environment, (b) an MDT meeting, and how might it be presented? E.g. line graphs, bar charts, raw tabulated data etc.

6. Could they be used to show longitudinal trends? Do you think they would be able to pick up ceiling effects or sensitive changes?

D. Barriers or facilitators

1. What do you think are the likely factors (barriers and facilitators) that may improve or discourage the completion of PROMs? IT issues/time? Importance of leadership during any implementation phase

2. Is there a risk we are going to overburden patients with multiple questionnaires? Have you heard of CAT/item banking?

For peer review only - http://bmjopen.bmj.com/site/about/guidelines.xhtml

3. What is the best way to implement into your workflow – could they affect your work flow patterns?

4. Would you want educating about PROMs – what form would that learning take? What specifically would you want more information on? Theory behind collection of PROS, Core outcome sets? Measures available, how they may be used in routine care?
   Are there opportunities to undertake advanced communications training?

5. What do you think about are the key issues about PROM collection in different patient groups (e.g. older patients, patients of non-white ethnicity)?

E. Use of PROMs with patients undergoing Home HD

1. In your opinion, how do patients dialysing at home differ from those dialysing in satellite units? Are they better at 'coping', dealing with change or is it their support structures? Do you think there is utility measuring PROs with this group of patients in particular? Role of carers in this group, how much should they be involved in the process?

2. Would any of your answers differ for patients undergoing Home HD in terms of when, where, how often, who should review and provide feedback?

3. Do you think Home HD patients are more likely to engage with digital technology?

F. Use of PROMs in Research

1. Have you have been involved in PROM use for research purposes? do you know how the measurement tool was chosen? Do you think it captured outcomes that were relevant for the research project?

2. Do you think it would be a good idea to use core outcome sets for renal trials? Should outcomes and the associated PROM that collects them differ between use for routine clinical and research purposes?

3. Do you think any of the practical considerations change when you are collecting PROMs for research rather than as part of routine practice? i.e. how often and where they are completed

4. Are there any special considerations to be given to feedback when using PROMs in a research setting?

For peer review only

For peer review only - http://bmjopen.bmj.com/site/about/guidelines.xhtml

COREQ (COnsolidated criteria for REporting Qualitative research) Checklist

A checklist of items that should be included in reports of qualitative research. You must report the page number in your manuscript where you consider each of the items listed in this checklist. If you have not included this information, either revise your manuscript accordingly before submitting or note N/A.

Topic	Item No.	Guide Questions/Description	Reported on Page No.
**Domain 1: Research team and reflexivity**			
Personal characteristics			
Interviewer/facilitator	1	Which author/s conducted the interview or focus group?	
Credentials	2	What were the researcher's credentials? E.g. PhD, MD	
Occupation	3	What was their occupation at the time of the study?	
Gender	4	Was the researcher male or female?	
Experience and training	5	What experience or training did the researcher have?	
Relationship with participants			
Relationship established	6	Was a relationship established prior to study commencement?	
Participant knowledge of the interviewer	7	What did the participants know about the researcher? e.g. personal goals, reasons for doing the research	
Interviewer characteristics	8	What characteristics were reported about the inter viewer/facilitator? e.g. Bias, assumptions, reasons and interests in the research topic	
**Domain 2: Study design**			
Theoretical framework			
Methodological orientation and Theory	9	What methodological orientation was stated to underpin the study? e.g. grounded theory, discourse analysis, ethnography, phenomenology, content analysis	
Participant selection			
Sampling	10	How were participants selected? e.g. purposive, convenience, consecutive, snowball	
Method of approach	11	How were participants approached? e.g. face-to-face, telephone, mail, email	
Sample size	12	How many participants were in the study?	
Non-participation	13	How many people refused to participate or dropped out? Reasons?	
Setting			
Setting of data collection	14	Where was the data collected? e.g. home, clinic, workplace	
Presence of non-participants	15	Was anyone else present besides the participants and researchers?	
Description of sample	16	What are the important characteristics of the sample? e.g. demographic data, date	
Data collection			
Interview guide	17	Were questions, prompts, guides provided by the authors? Was it pilot tested?	
Repeat interviews	18	Were repeat inter views carried out? If yes, how many?	
Audio/visual recording	19	Did the research use audio or visual recording to collect the data?	
Field notes	20	Were field notes made during and/or after the inter view or focus group?	
Duration	21	What was the duration of the inter views or focus group?	
Data saturation	22	Was data saturation discussed?	
Transcripts returned	23	Were transcripts returned to participants for comment and/or	

For peer review only

Topic	Item No.	Guide Questions/Description	Reported on Page No.
		correction?	
**Domain 3: analysis and findings**			
Data analysis			
Number of data coders	24	How many data coders coded the data?	
Description of the coding tree	25	Did authors provide a description of the coding tree?	
Derivation of themes	26	Were themes identified in advance or derived from the data?	
Software	27	What software, if applicable, was used to manage the data?	
Participant checking	28	Did participants provide feedback on the findings?	
Reporting			
Quotations presented	29	Were participant quotations presented to illustrate the themes/findings? Was each quotation identified? e.g. participant number	
Data and findings consistent	30	Was there consistency between the data presented and the findings?	
Clarity of major themes	31	Were major themes clearly presented in the findings?	
Clarity of minor themes	32	Is there a description of diverse cases or discussion of minor themes?	

Developed from: Tong A, Sainsbury P, Craig J. Consolidated criteria for reporting qualitative research (COREQ): a 32-item checklist for interviews and focus groups. *International Journal for Quality in Health Care*. 2007. Volume 19, Number 6: pp. 349 – 357

Once you have completed this checklist, please save a copy and upload it as part of your submission. DO NOT include this checklist as part of the main manuscript document. It must be uploaded as a separate file.

For peer review only
